# Pregnancy-Associated Changes in Pharmacokinetics: A Systematic Review

**DOI:** 10.1371/journal.pmed.1002160

**Published:** 2016-11-01

**Authors:** Gali Pariente, Tom Leibson, Alexandra Carls, Thomasin Adams-Webber, Shinya Ito, Gideon Koren

**Affiliations:** 1 Division of Clinical Pharmacology and Toxicology, Hospital for Sick Children, Toronto, Ontario, Canada; 2 Hospital Library, Hospital for Sick Children, Toronto, Ontario, Canada; 3 Research Institute, Hospital for Sick Children, Toronto, Ontario, Canada; 4 Department of Paediatrics, University of Toronto, Toronto, Ontario, Canada; 5 Department of Pharmacology & Toxicology, University of Toronto, Toronto, Ontario, Canada; 6 Leslie Dan Faculty of Pharmacy, University of Toronto, Toronto, Ontario, Canada; PLOS Medicine, UNITED KINGDOM

## Abstract

**Background:**

Women are commonly prescribed a variety of medications during pregnancy. As most organ systems are affected by the substantial anatomical and physiological changes that occur during pregnancy, it is expected that pharmacokinetics (PK) (absorption, distribution, metabolism, and excretion of drugs) would also be affected in ways that may necessitate changes in dosing schedules. The objective of this study was to systematically identify existing clinically relevant evidence on PK changes during pregnancy.

**Methods and Findings:**

Systematic searches were conducted in MEDLINE (Ovid), Embase (Ovid), Cochrane Central Register of Controlled Trials (Ovid), and Web of Science (Thomson Reuters), from database inception to August 31, 2015. An update of the search from September 1, 2015, to May 20, 2016, was performed, and relevant data were added to the present review. No language or date restrictions were applied. All publications of clinical PK studies involving a group of pregnant women with a comparison to nonpregnant participants or nonpregnant population data were eligible to be included in this review. A total of 198 studies involving 121 different medications fulfilled the inclusion criteria. In these studies, commonly investigated drug classes included antiretrovirals (54 studies), antiepileptic drugs (27 studies), antibiotics (23 studies), antimalarial drugs (22 studies), and cardiovascular drugs (17 studies). Overall, pregnancy-associated changes in PK parameters were often observed as consistent findings among many studies, particularly enhanced drug elimination and decreased exposure to total drugs (bound and unbound to plasma proteins) at a given dose. However, associated alterations in clinical responses and outcomes, or lack thereof, remain largely unknown.

**Conclusion:**

This systematic review of pregnancy-associated PK changes identifies a significant gap between the accumulating knowledge of PK changes in pregnant women and our understanding of their clinical impact for both mother and fetus. It is essential for clinicians to be aware of these unique pregnancy-related changes in PK, and to critically examine their clinical implications.

## Abstract 2

Shinya Ito and colleagues systematically review the literature to identify research reporting medications reporting pregnancy-associated changes in pharmacokinetics.

## Author Summary

**Why Was This Study Done:**

- Pregnant women take a variety of medications, including prescription and over-the-counter medications, with an estimated prevalence of greater than 90%.
- Some studies have demonstrated significant changes in pharmacokinetics (absorption, distribution, metabolism, and excretion of drugs) during pregnancy and resultant clinical impact, but others have not, which calls for critical assessment of the evidence.

**What Did the Researchers Do and Find?:**

- We conducted a systematic review, and identified 198 studies, involving 121 different medications, that fulfilled the inclusion criteria.
- Decrease in drug exposure mainly due to increased elimination was frequently observed across the drug classes.
- There is a lack of studies describing changes in clinical outcomes, or the lack thereof, associated with altered pharmacokinetics during pregnancy.

**What Do These Findings Mean?:**

- A significant gap exists between our knowledge of pharmacokinetic changes in pregnancy and their clinical consequences.
- It is essential for clinicians to be aware of these pregnancy-related changes in pharmacokinetics, and to critically examine their potential clinical implications.

## Introduction

Women frequently take a variety of medications during pregnancy, including prescription, over-the-counter (OTC), and herbal agents [1,2]. During the last three decades the average number of medications (prescription and nonprescription) used per woman in North America during the first trimester increased by 60% from 1.6 to 2.6 [3]. More recently, from 2006 to 2008, over 80% of women reported using at least one medication during the first trimester, and over 90% reported using at least one medication at any point during their pregnancy [3]. Other studies have demonstrated increased rates of use of various OTC medications in the first, second, or third trimester of pregnancy compared to the prepregnancy period [4]. While some studies have found that the proportion of women receiving at least one prescription medicine increases from the first to third trimester of pregnancy [5,6], others have found that rates of prescription drug use are highest in the first trimester of pregnancy [1,7]. The most common medications used in pregnancy are nonprescription or OTC medications [4]. A longitudinal study aimed at identifying the medications that are most often consumed during pregnancy demonstrated that 95.8% of participants took prescription medications, 92.6% self-medicated with OTC medications, and 45.2% used herbal medications [2].

Most organ systems are affected by substantial anatomical and physiological changes during pregnancy. Such pregnancy-related changes are observed in decreased gastrointestinal motility and increased gastric pH (impacting absorption), increased total body water and plasma volume and decreased concentrations of drug-binding proteins (affecting the apparent volume of distribution and, in some cases, clearance rates), increased glomerular filtration rate (increasing renal clearance), and altered activity of drug-metabolizing enzymes in the liver (affecting hepatic clearance). Overall, these changes in physiological indices take place progressively during gestation (reviewed in [8] and [9]). The increases in cardiac output, total body water, fat compartment, and glomerular filtration rate, together with the decrease in plasma albumin concentration and altered activity of drug-metabolizing enzymes, are all reported to peak during the third trimester (reviewed in [8] and [10]). Table 1 presents typical pregnancy-related changes in organ function leading to altered pharmacokinetics (PK) [10–16]. Changes during pregnancy in drug metabolism by cytochrome P450 isoenzymes (i.e., CYP3A4, CYP2D6, CYP2C9, CYP1A2, and CYP2C19) and by uridine 5′-diphospho-glucuronosyltransferase (UGT) isoenzymes (i.e., UGT1A4 and UGT2B7) have also been demonstrated (Table 2) [10,17–20].

**Table 1:** Physiological changes during pregnancy: effects on drug disposition [10–16].

Parameter	Consequences
Delayed gastric emptying and increased gastric pH	Altered drug bioavailability and delayed time to peak levels after oral administration
Increased cardiac output	Increased hepatic blood flow; increased elimination for some drugs
Increased total body water, extracellular fluid	Altered drug disposition; increased *V*_d_ for hydrophilic drugs
Increased fat compartment	Decreased elimination of lipid-soluble drugs; increased *V*_d_ for hydrophobic drugs
Increased renal blood flow and glomerular filtration rate	Increased renal clearance
Decreased plasma albumin concentration	Increased free fraction of drug
Altered CYP450 and UGT activity	Altered oral bioavailability and hepatic elimination

UGT, uridine diphosphate glucuronosyltransferase; *V*_d_, volume of distribution.

**Table 2:** Reported effects of pregnancy on hepatic enzyme activity.

Enzyme	Effect of Pregnancy [Reference]	Substrate Examples
CYP1A2	Decreased [18]	Paracetamol, propranolol, theophylline
CYP2B6	Increased [21]	Methadone, efavirenz, sertraline
CYP2C8	Increased [22]	Verapamil, fluvastatin
CYP2C9	Increased [23,24]	Glyburide, phenytoin
CYP2C19	Decreased [23,25]	Proguanil, indomethacin, citalopram, escitalopram
CYP2D6	Increased [17]	Alprenolol, codeine, fluoxetine
CYP2E1	Increased [26]	Disulfiram, theophylline
CYP3A4	Increased [27]	Darunavir, citalopram
Uridine 5′-diphospho-glucuronosyltransferases	Increased [28]	Lamotrigine, morphine

For some drug classes, a large number of PK clinical trials during pregnancy are available in the literature [29–34]. A recent review noted that, since 2008, about a third of these trials investigated drugs used in the treatment of acute labor and delivery issues, another third investigated drugs used in infectious disease treatment during pregnancy, and the remaining third investigated drugs used for various antepartum indications [35]. However, for the large majority of drugs used during pregnancy, there is little or no information available regarding PK changes or dosage requirements during pregnancy [35]. Moreover, it is often unclear if observed PK changes lead to alterations in drug efficacy and/or adverse effect profiles. Given the complexity of the field, the lack of clear understanding of the clinical significance of PK changes, and renewed recognition of the need to rationalize drug therapy for pregnant and lactating women, it is imperative to systematically examine existing data on PK changes in pregnancy and their potential clinical impact.

The objective of this study was to systematically identify all existing evidence of PK changes during pregnancy in the context of clinical significance. We hypothesized that known physiological changes occurring during pregnancy and associated PK alterations could consequently be translated into changes in dosing guidelines.

## Methods

This research involved a structured review of the literature, according to the PRISMA guidelines [36] (S1 Checklist).

### Search Strategy

Searches were conducted in MEDLINE (Ovid), Embase (Ovid), Cochrane Central Register of Controlled Trials (Ovid), and Web of Science (Thomson Reuters) from database inception to August 31, 2015 (S1 Table). An update of the search from September 1, 2015, to May 20, 2016, was performed, and relevant data were retrieved and added to the review (S2 Table). Text words and, where applicable, database subject heading fields (e.g., MeSH) were used for the following concepts: pregnancy AND pharmacokinetics OR dosing OR clearance OR distribution OR absorption OR metabolism OR excretion OR Cmax OR Tmax OR Ctrough OR AUC OR Vd OR t1/2 OR protein binding AND specific study types (randomized controlled trial, non-randomized controlled clinical trial, cohort study, case–control study, or case series). Truncation symbols were used with the text words, when appropriate, to capture variations in spelling and word endings. Subsequently, we reviewed the identified studies and examined their references to identify further potential articles. Information available from relevant conferences was also reviewed. No publication date, language, or location restrictions were applied.

### Study Selection

In order to locate all published literature, we established a set of criteria to define types of studies to be reviewed. Inclusion criteria were as follows: (1) the study reported dosing data or at least one PK parameter of interest in pregnant women; (2) a comparison of the dosing data or PK parameter between pregnant and nonpregnant women was done; and (3) the data are described in the form of a peer-reviewed randomized controlled trial, non-randomized controlled clinical trial, cohort study, case–control study, or case series. The review did not cover animal studies, case reports, or studies containing no original research or data. Retrieved articles were inspected by two independent reviewers (G. P. and T. L.) to determine whether they met the inclusion criteria. In cases where the eligibility of the study was unclear, it was reviewed by a third independent reviewer (G. K.). The full texts were retrieved and read in full.

### Data Extraction

The data extractors (G. P. and T. L.) reviewed each of the included studies independently and extracted data according to the predetermined guidelines, using a predesigned data extraction form. When needed, authors of the included studies were contacted for missing data; however, none of the authors who were contacted for more information responded. Data from studies presented in multiple publications were identified to avoid duplications and were reported as a single study, with all other relevant publications listed.

### Data Presentation and Analysis

#### Results of the literature search

**Fig 1:**
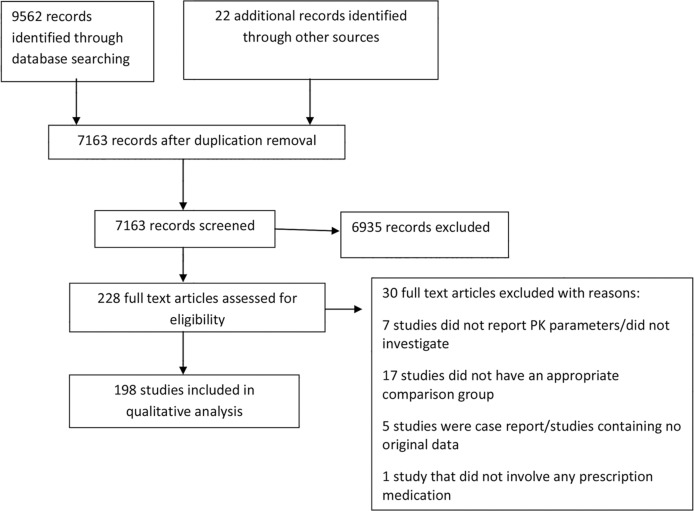
Flow diagram of numbers of studies screened, assessed for eligibility, and included in the review. PK, pharmacokinetics.

The results from each step of the review process are documented in a PRISMA flow diagram (Fig 1), with an overall summary of the number and types of articles included in the review.

When more than one study reported the same PK parameter(s) for the same drug, these parameters were examined for consistency in the change direction (i.e., decrease, increase, or no change). When study data were presented by trimester, the PK parameters obtained during the third trimester were selected for this study because the majority of the pregnancy-associated physiological changes peak during the third trimester.

Drugs were divided into two major categories according to between-study agreement of directions of statistically significant changes in PK parameters. If statistically significant pregnancy-associated changes in PK parameters were in the same direction (e.g., increase in clearance and decrease in volume of distribution) among the studies for all reported PK parameters, we categorized the drug as “consistent.” On the other hand, a drug was categorized as “inconsistent” if at least one study reported a statistically significant change in a PK parameter in the opposite direction (e.g., increased Cl in one study and decreased Cl in the other). The potential source of inconsistency is speculated on and addressed in the Discussion. Note that the definition of the categories described above is based on statistically significant changes of PK parameters, but statistically non-significant changes are also presented, for completeness. In addition, if only one study showed a statistically significant PK parameter change for a drug, the drug was included in the “consistent” category for simplicity of the data presentation, even though the PK parameters were reported in only one study.

#### Quality assessment

The quality of each accepted article was assessed using the ClinPK checklist [37] for assessing methodological quality in clinical PK studies (Table 3).

**Table 3:** ClinPK checklist for assessing methodological quality in clinical pharmacokinetic studies [37].

Section	Checklist Item Number	Checklist Item
**Title/abstract**	1	The title identifies the drug(s) and patient population(s) studied.
	2	The abstract minimally includes the name of the drug(s) studied, the route of administration, the population in whom it was studied, and the results of the primary objective and major clinical pharmacokinetic findings.
**Background**	3	Pharmacokinetic data (i.e., absorption, distribution, metabolism, excretion) that [are] known and relevant to the drugs being studied [are] described.
	4	An explanation of the study rationale is provided.
	5	Specific objectives or hypotheses [are] provided.
**Methods**	6	Eligibility criteria of study participants are described.
	7	Co-administration (or lack thereof) of study drug(s) with other potentially interacting drugs or food within this study is described.
	8	Drug preparation and administration characteristics including dose, route, formulation, infusion duration (if applicable), and frequency are described.
	9	Body fluid or tissue sampling (timing, frequency, and storage) for quantitative drug measurement is described.
	10	Validation of quantitative bioanalytical methods used in the study [is] referenced or described if applicable.
	11	Pharmacokinetic modeling methods and software used are described, including assumptions made regarding the number of compartments and order of kinetics (zero, first, or mixed order).
	12	For population pharmacokinetic studies, covariates incorporated into pharmacokinetic models are identified and described.
	13	Formulas for calculated variables (such as creatinine clearance, body surface area, AUC, and adjusted body weight) are provided or referenced.
	14	The specific body weight used in drug dosing and pharmacokinetic calculations [is] reported (i.e., ideal body weight versus actual body weight versus adjusted body weight).
	15	Statistical methods including software used are described.
**Results**	16	Study withdrawals or subjects lost to follow-up (or lack thereof) are reported.
	17	Quantification of missing or excluded data is provided if applicable.
	18	All relevant variables that may explain inter- and intra-patient pharmacokinetic variability (including: age, sex, end-organ function, ethnicity, weight or BMI, health status or severity of illness, and pertinent co-morbidities) are provided with appropriate measures of variance.
	19	Results of pharmacokinetic analyses are reported with appropriate measures of precision (such as range or 95% confidence intervals).
	20	Studies in patients receiving extracorporeal drug removal (i.e., dialysis) should report the mode of drug removal, type of filters used, duration of therapy, and relevant flow rates.
	21	In studies of drug bioavailability comparing two formulations of the same drug, F (bioavailability), AUC, Cmax (maximal concentration), and Tmax (time to maximal concentration) should be reported.
**Discussion/conclusion**	22	Study limitations describing potential sources of bias and imprecision where relevant should be described.
	23	The relevance of study findings (applicability, external validity) is described.
**Other information**	24	Funding sources and conflicts of interest for the authors are disclosed.

All the items presented in the table correspond to the original checklist as published in [37].

AUC, area under the curve; BMI, body mass index.

No discrepancies exist between the original protocol and the final data analyses.

## Results

### Literature Retrieval

The search strategy for the comprehensive systematic review retrieved 9,562 articles, and after removing duplicates, the first screen on title and abstract was performed on 7,163 articles (Fig 1). For 6,935 of these, the title or abstract clearly indicated that the topic of the article was not relevant to the review question or did not satisfy one of the inclusion criteria. The remaining 228 articles were screened using the full text, applying the full set of eligibility criteria. After applying the eligibility criteria, 202 articles containing comparisons of PK parameters of different drugs between pregnant and nonpregnant women were eligible for inclusion. Twenty-six studies were excluded because they didn’t report PK parameters, didn’t include a comparison group, or were either review papers or case reports (S3 Table). Following review, four further articles were excluded because they duplicated the same outcome domain, in the same cohort, as another article. The remaining 198 articles were included in the data extraction for the comprehensive systematic review. Twenty-two additional articles were identified using a monthly update search between September 1, 2015, and May 20, 2016. Hence, this review article summarizes the results of a total of 198 studies, involving 121 different medications, reporting comparisons of different PK parameters and dosing data between pregnant and nonpregnant cohorts.

Reviewed studies were found to vary widely in both design and quality (S4 Table). There were some differences in the stages of pregnancy in which the women were investigated; while most of the studies provided third trimester results, others reported results from both the second and the third trimesters together [38–42] or separately [43–46], and a few reported results from all trimesters together [47] or separately [48]. Two studies reported only first trimester results [49,50].

### Studies Comparing Pregnant and Nonpregnant Women for Each Drug Class

**Fig 2:**
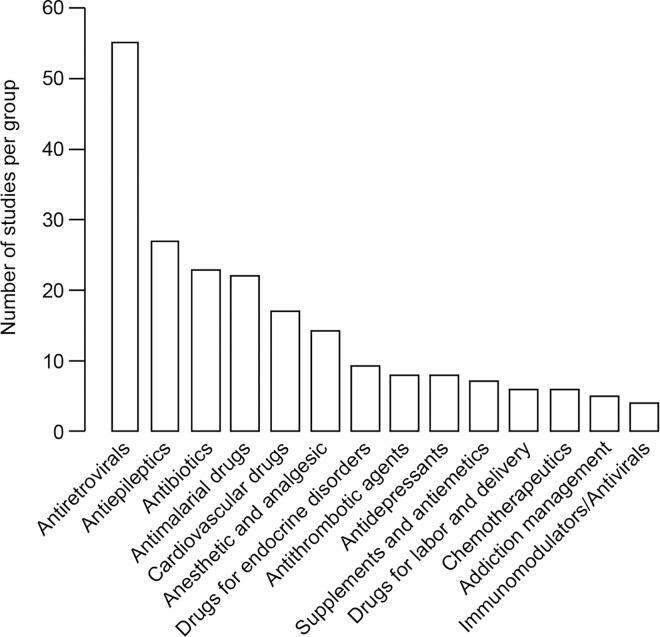
Number of studies comparing pregnant and nonpregnant women for each drug class.

Certain drug classes were far more commonly investigated during pregnancy than others (Fig 2). Approximately one-half of the studies (48%) addressed medications given chronically during pregnancy. Of the studies of chronic medications, 54 studies focused on drugs for HIV treatment and vertical transmission prevention, 27 studies focused on antiepileptic drugs, 17 studies focused on drugs related to cardiovascular disorders, and nine studies focused on drugs for endocrine disorders. An additional eight studies investigated antidepressants and anxiolytic drugs, five other studies focused on drugs involved in addiction management, and two studies described drugs treating immunological conditions. In comparison, 84 studies addressed drugs used in the treatment of acute issues during pregnancy; among them, 23 studies addressed antibiotics, 22 studies addressed antimalarial medications, 13 studies addressed analgesics or anesthetic drugs, and eight studies addressed antithrombotic drugs in pregnancy. Fifty-one studies investigated more than one drug. Among the antiretroviral class, all studies but one presented women living with HIV infection who were treated with more than one antiretroviral medication. Eleven of 22 studies investigating antimalarial drugs described more than one drug given to the same patient population. Four of 27 studies investigating antiepileptic drugs described more than one drug given to the same patient population. Other drug classes that reported results of pregnant women taking more than one drug included antibiotics (four studies), anesthesia and analgesia drugs (one study), and antiemetics (one study).

### Reported Pharmacokinetics Parameters

PK parameters of interest as defined by our search terms were the following: elimination half-life (*t*_1/2_), clearance (Cl), *C*_max_, *C*_trough_, concentration-to-dose ratio (C/D ratio), area under the curve (AUC), volume of distribution (*V*_d_), and protein binding (i.e., free fraction). The majority of the studies reported on various combinations of some of these PK parameters of interest (Table 4). The most frequently reported PK parameter was Cl, followed by AUC, *t*_1/2_, and *C*_max_ with 116, 103, 88, and 87 counts, respectively. In most of the studies that focused on the free fraction of a drug in plasma, the free fraction was the only PK parameter reported in the study. While Cl and AUC were the most frequently reported parameters, both parameters were reported for only 46% of the drugs. Whereas more than half of the drugs (53%) were described with both the Cl and the *t*_1/2_, only 16% of the drugs included *C*_trough_, *C*_max_, and AUC. The latter group mostly consisted of antiretroviral drugs. *C*_max_ and AUC were described together for 30% of the drugs.

**Table 4:** Pharmacokinetics parameters—data count.

Category	PK Parameter	Number of Studies
**Dose independent**	*t*_1/2_ (elimination half-life)	88
	Cl (clearance)	116
**Dose dependent**	*C*_trough_	48
	*V*_d_ (volume of distribution)	62
	*T*_max_	63
	*C*_max_	87
	AUC (area under the curve)	103
	Free fraction in plasma	15

### Pharmacokinetics Parameters That Are Vital for Dosing Decision Support in Pregnancy

We clustered the different PK parameters into three groups. (1) Distribution parameters are *V*_d_ and percent of free fraction. *V*_d_ defines how widely the drug is spread in the body. Larger *V*_d_ causes lower peak plasma concentration (*C*_max_) and also longer elimination half-life. Percent free fraction represents the fraction (percent) of the drug in plasma that is unbound to plasma proteins and, therefore, likely to be pharmacologically active. (2) Exposure parameters are *C*_max_, *C*_trough_, AUC, C/D ratio. These represent indices of plasma drug concentrations. *C*_max_ and *C*_trough_ are the highest and lowest levels within a dosing interval, respectively. AUC is literally the area bounded by the drug concentration–time curve and the *x*-axis, equivalent to an average drug concentration over time. C/D ratio is the dose-standardized drug concentration in plasma or serum at a given time. By and large, these parameters signify drug exposure levels at a given time point or on average, thereby potentially serving as a surrogate for drug effects. (3) Elimination parameters are *t*_1/2_ and clearance. Half-life is related to the velocity of a drug’s disappearance from plasma/serum. Clearance is an index of drug elimination capacity: higher clearance results in a smaller AUC and a shorter elimination half-life, reducing drug exposure levels.

Tables 5–18 provide information regarding changes in PK parameters (weight-standardized values, if available) during pregnancy compared to the nonpregnant state, assorted by drug classes and the data agreement definitions provided above. In these tables, non-significant results are shown together with statistically significant results (in bold). When a certain PK parameter was reported by several studies, the median value and the range in parentheses are provided. The quality column represents the quality score that was assigned to the study, according to the ClinPK Statement checklist. If the drug was investigated in more than one study, the quality column presents the average quality score of all the studies. Among the frequently investigated drug classes (antibiotics, antidepressants, antiepileptics, cardiovascular drugs, antiretrovirals, and antimalarials), studies have demonstrated enhanced elimination together with a decrease in exposure in pregnancy, indicating decreased availability of the drugs in pregnant women compared to nonpregnant women so far as total drug levels (bound plus unbound) are concerned.

**Table 5:** Antibiotics: consistent/single studies of pregnancy-associated pharmacokinetic changes (percent calculated as pregnant/nonpregnant values).

Drug [Reference]	Number of Studies	Total Number of Women (Nonpregnant/Pregnant)	Average Quality (24 Items)	Distribution Parameters	Exposure Parameters	Elimination Parameters	Trimester
Amoxicillin [43]	1	16/16	22	NR	NR	**Cl 140%, *t***_**1/2**_ **81%**	3rd
Azithromycin [47,51]	2	54/84	19.5	***V***_**d**_ **121%** ^**&**^	AUC 90%^**&**^	*t*_1/2_ 101%^**&**^	1st–3rd
Cefatrizine [52]	1	20/20	19	NR	***C***_**max**_ **55%, AUC 57%**	***t***_**1/2**_ **163%**	2nd
Cefazolin [39,53,54]	3	10^$^/54	18.6	*V*_d_ 80% (72%–89%)^**&**^, **free fraction 131%** ^**&**^	AUC 68%^**&**^	Cl 102% (65%–140%)^**&**^, ***t***_**1/2**_ **65%** ^**&**^, *t*_1/2_ 131%^**&**^	2nd–3rd
Cefoperazone [55]	1	9/11	13	**Free fraction 208%**	NR	NR	3rd
Cefradine [54]	1	12/12	19	*V*_d_ 113%	**AUC 62%**	**Cl 154%, *t***_**1/2**_ **73%**	1st–3rd
Ceftazidime [56]	1	12/12	16	NR	NR	**Cl 165%**	3rd
Cefuroxime [57]	1	7/7	13	*V*_d_ 109%	**AUC 69%**	**Cl 142%, *t***_**1/2**_ **75%**	1st–3rd
Cloxacillin [48,58]	2	14/33	13.5	**Free fraction 154% (146%–162%)**	NR	NR	3rd
Flucloxacillin [58]	1	7/22	11	**Free fraction 148%**	NR	NR	3rd
Imipenem [59]	1	6/7	15	***V***_**d**_ **249%**	***C***_**max**_ **34%, AUC 41%**	**Cl 287%,** *t*_1/2_ 87%	3rd
Mecillinam [60]	1	6/10	17	***V***_**d**_ **224%**	*C*_max_ 85%, AUC 85%	Cl 103%, ***t***_**1/2**_ **142%**	3rd
Moxifloxacin [61]	1	9/6	11	***V***_**d**_ **329%**	***C***_**max**_ **31%, AUC 21%**	***t***_**1/2**_ **63%**	3rd
Penicillin V [62]	1	6/6	16	NR	*C*_max_ 96%, **AUC 60%**	Cl 118%, ***t***_**1/2**_ **30%**	3rd
Piperacillin [63–65]	3	11/18	12.3	***V***_**d**_ **161%,** *V*_d_ 145% (136%–155%)	***C***_**max**_ **50%** ^**&**^, *C*_max_ 57%^**&**^, **AUC 61%** ^**&**^, AUC 110%^**&**^	**Cl 284%**, Cl 130% (96%–165%), *t*_1/2_ 86% (70%–135%)	3rd
Trimethoprim [66]	1	8/10	11	***V***_**d**_ **407%**	NR	**Cl 346%,** *t*_1/2_ 100%	2nd–3rd
Tazobactam [64]	1	6/5	13	***V***_**d**_ **150%**	*C*_max_ 75%, AUC 106%	***t***_**1/2**_ **156%**	3rd

Significant results are marked in bold.

^&^Parameter not reported in all studies.

^$^Comparison group in one study is published data.

NR, not reported.

**Table 6:** Antibiotics: inconsistent studies of pregnancy-associated pharmacokinetic changes (percent calculated as pregnant/non-pregnant values).

Drug [Reference]	Number of Studies	Total Number of Women (Nonpregnant/Pregnant)	Average Quality	Distribution Parameters	Exposure Parameters	Elimination Parameters	Potential Sources for Inconsistency	Trimester
Ampicillin [67,68]	2	32/35	11.5	*V*_d_ 96%^&^	*C*_trough_ 108%^&^, **AUC 79%** ^&^	**Cl 122%** ^&^ **, inconsistent data for *t***_**1/2**_ ^#^	Comparison group selection	3rd

Significant results are marked in bold.

^&^Parameter reported in one study.

^#^Numbers not provided.

**Table 7:** Antidepressant/anxiolytic drugs: consistent/single studies of pregnancy associated pharmacokinetic changes (percent calculated as pregnant/nonpregnant values).

Drug [Reference]	Number of Studies	Total Number of Women (Nonpregnant/Pregnant)	Average Quality	Distribution Parameters	Exposure Parameters	Elimination Parameters	Trimester
Citalopram [69,70]	2	16/16	15	NR	***C***_**trough**_ **59%** ^**&**^	NR	3rd
Fluoxetine [71]	1	11/8	16	NR	***C***_**trough**_ **39%**	NR	3rd
Paroxetine [72]	1	12/12	11	NR	**Lower concentrations** ^**β**^	NR	3rd
Venlafaxine [73])	1	7/7	16	NR	**Concentrations 87%**	NR	3rd
Clorazepate [74]	1	7/7	17	NR	***C***_**max**_ **51%**	**Cl 209%,** *t*_1/2_ 50%	3rd
Midazolam [75,76]	2	23/21	18	***V***_**d**_ **112%** ^**&**^ **, free fraction 163%** ^**&**^	***C***_**max**_ **68%** ^**&**^ **, AUC 53%** ^**&**^, AUC 62%^**&**^	**Cl 184% (159%–210%),** *t*_1/2_ 87% (79%–96%)	3rd

Significant results are marked in bold.

^&^Parameter not reported in all studies.

^β^Numbers were not provided.

NR, not reported.

**Table 8:** Antiepileptic drugs: consistent/single studies of pregnancy associated pharmacokinetic changes (percent calculated as pregnant/nonpregnant values).

Drug Reference]	Number of Studies	Total Number of Women (Nonpregnant/Pregnant)	Average Quality (24 Items)	Distribution Parameters	Exposure Parameters	Elimination Parameters	Trimester
Carbamazepine [77–85]	9	128/130	11.7	**Free fraction 116% (113%–119%)** ^**&**^ **, free** fraction 101% (95%–107%)^**&**^	**Total concentration 79%** ^**&**^	**Cl 127% (116%–140%)** ^**&**^, Cl 110% (108%–112%)^**&**^	1st–3rd
Lamotrigine [83,86–93]	9	208/241	15.7	NR	**C/D ratio 34%** ^**&**^	**Cl 212% (185%–240%)** ^**&**^	3rd
Levetiracetam [16,83,94,95]	4	47/47	14	NR	**C/D ratio 45% (39%–52%)** ^**&**^	**Cl 269% (197%–342%)** ^**&**^	3rd
Oxcarbazepine [83,96–98]	4	28/28	13.7	NR	**Lower concentration and C/D ratio** ^**&**^ ^,^ ^β^	Cl 237%^**&**^	3rd
Phenytoin [81,82,84,99]	4	82/78	12.5	**Free fraction 126%** ^**&**^	**Total concentration 67% (51%–84%)** ^**&**^	**Cl 145% (130%–160%)** ^**&**^	1st–3rd
Phenobarbital [81]	1	11/11	9	**Free fraction 112%**	**Total concentration 53%**	**Cl 125%**	3rd
Topiramate [83,100,101]	3	21/25	16	NR	**C/D ratio 60% (57%–64%)** ^**&**^	Cl 110%^**&**^	3rd

Significant results are marked in bold.

^&^Parameter not reported in all studies.

^β^Numbers were not provided.

NR, not reported.

**Table 9:** Drugs for analgesia and anesthesia: consistent/single studies of pregnancy-associated pharmacokinetic changes (percent calculated as pregnant/nonpregnant values).

Drug [Reference]	Number of Studies	Total Number of Women (Nonpregnant/Pregnant)	Average Quality (24 Items)	Distribution Parameters	Exposure Parameters	Elimination Parameters	Trimester
Ketorolac [102]	1	8/8	16	***V***_**d**_ **134%**	NR	**Cl 150%,** *t*_1/2_ 108%	3rd
Morphine [103]	1	6/8	19	*V*_d_ 92%	**AUC 96%**	**Cl 169%, *t***_**1/2**_ **51%**	3rd
Paracetamol [49,102,104–107]	6	52/85	18.1	***V***_**d**_ **182%** ^**&**^	***C***_**trough**_ **56%** ^**&**^, *C*_max_ 87% (42%–96%)^**&**^, **AUC 72%** ^**&**^, AUC 83%^**&**^	**Cl 142% (132%–196%), *t***_**1/2**_ **80%** ^**&**^, *t*_1/2_ 95% (72%–119%^**&**^	1st + 3rd

Significant results are marked in bold.

^&^Parameter not reported in all studies.

NR, not reported.

**Table 10:** Drugs for analgesia and anesthesia: inconsistent studies of pregnancy-associated pharmacokinetic changes (percent calculated as pregnant/nonpregnant values).

Drug [Reference]	Number of Studies	Total Number of Women (Nonpregnant/Pregnant)	Average Quality	Distribution Parameters	Exposure Parameters	Elimination Parameters	Potential Sources for Inconsistency	Trimester
Propofol [108–110]	3	22/26	15	*V*_d_ 88% (79%–98%)^&^	*C*_max_ 141%)^&^	**Inconsistent data for Cl** ^&^ ^,^ ^#^, *t*_1/2_ 80.5% (80%–81%)^&^	Different sampling period	3rd

Significant results are marked in bold.

^&^Parameter not reported in all studies

^#^Number not provided.

**Table 11:** Antithrombotic drugs: consistent/single studies of pregnancy-associated pharmacokinetic changes (percent calculated as pregnant/nonpregnant values).

Drug [Reference]	Number of Studies	Total Number of Women (Nonpregnant/Pregnant)	Average Quality (24 Items)	Distribution Parameters	Exposure Parameters	Elimination Parameters	Trimester
Antipyrine [111]	1	6/4	13	NR	NR	**Cl 242%, *t***_**1/2**_ **44%**	3rd
Aspirin [112]	1	11/10	18	NR	***C***_**max**_ **68%,** AUC 76%	NR	3rd

Significant results are marked in bold.

NR, not reported.

**Table 12:** Antithrombotic drugs: inconsistent studies of pregnancy-associated pharmacokinetic changes.

Drug [Reference]	Number of Studies	Total Number of Women (Nonpregnant/Pregnant)	Average Quality	Distribution Parameters	Exposure Parameters	Elimination Parameters	Potential Sources for Inconsistency	Trimester
Heparin [113,114]	2	12/12	17	NR	*C*_trough_ 400%^**&**^, **inconsistent data for *C***_**max**_ **and AUC** ^**#**^	Cl 72%^**&**^	Different population (healthy versus non-healthy pregnant women), different dosing regimens	2nd–3rd
Low-molecular-weight heparin [46,114–117]	5	86/134	15.8	***V***_**d**_ **119%** ^**&**^, *V*_d_ 162%^**&**^	*C*_trough_ 300%^**&**^, **inconsistent data for *C***_**max**_ **and AUC** ^**#**^ (equivalent to anti-Xa activity)	**Cl 133% (117%–150%)** ^**&**^, Cl 219%^**&**^	Different underlying disease, prophylactic versus therapeutic doses, different time points of blood sampling	3rd

Significant results are marked in bold.

^&^Parameter not reported in all studies.

^#^Number not provided.

NR, not reported.

**Table 13:** Cardiovascular drugs: consistent/single studies of pregnancy-associated pharmacokinetic changes (percent calculated as pregnant/nonpregnant values).

Drug [Reference]	Number of Studies	Total Number of Women (Nonpregnant/Pregnant)	Average Quality (24 Items)	Distribution Parameters	Exposure Parameters	Elimination Parameters	Trimester
Atenolol [118,119]	2	27/27	18.5	NR	*C*_max_ 93%^&^, AUC 96%^&^	**Cl 131%** ^&^ **, *t***_**1/2**_ **85%** ^&^	3rd
Clonidine [120]	1	0^!^/17	16	NR	NR	**Cl 179%**	3rd
Digoxin [75]	1	12/12	18	**Free fraction 106%**	*C*_max_ 72%, **AUC 78%**	**Cl 157%,** *t*_1/2_ 82%	3rd
Fenoterol [121]	1	5/9	15	***V***_**d**_ **58%**	NR	Cl 93%	2nd–3rd
Furosemide [122]	1	NR/9	11	***V***_**d**_ **188%**	***C***_**max**_ **41%**	**Cl 165%,** *t*_1/2_ 111%	3rd
Labetalol [123–125]	3	64/75	18	**Higher *V***_**d**_ ^#^ ^,^ ^&^, *V*_d_ 58%^&^	NR	**Higher Cl** ^#^ ^,^ ^&^, Cl 71%^&^, *t*_1/2_ 96%	3rd
Metildigoxin [126]	1	1/8	14	NR	NR	**Cl 130%**	3rd
Metoprolol [127]	1	8/8	17	NR	**Concentration 25%**	NR	3rd
Nifedipine [128]	1	0^!^/15	15	NR	***C***_**max**_ **52%**	**Cl 408%, *t***_**1/2**_ **37%**	3rd
Penbutolol [40]	1	10/11	13	**Free fraction 114%**	NR	NR	2nd–3rd
Sotalol [129]	1	6/6	18	*V*_d_ 108%	AUC 60%	**Cl 160%,** *t*_1/2_ 70%	3rd

Significant results are marked in bold.

^&^Parameter not reported in all studies.

^!^Data compared to published reports.

^#^Numbers not provided.

NR, not reported.

**Table 14:** Antiretroviral drugs: consistent/single studies of pregnancy-associated pharmacokinetic changes (percent calculated as pregnant/nonpregnant values).

Drug Reference]	Number of Studies	Total Number of Women (Nonpregnant/Pregnant)	Average Quality (24 Items)	Distribution Parameters	Exposure Parameters	Elimination Parameters	Trimester
Abacavir [130]	1	25/25	19	NR	***C***_**max**_ **90%,** AUC 109%	Cl 91%, *t*_1/2_ 102%	3rd
Atazanavir [131–137]	7	292/287	18.4	*V*_d_ 74% (73%–154%)^&^	***C***_**trough**_ **54% (43%–66%)** ^&^, *C*_trough_ 79% (49%–132%), ***C***_**max**_ **65% (60%–90%)** ^&^, *C*_max_ 73% (63%–99%)^&^, **AUC 72% (64%–73%)** ^&^, AUC 78% (62%–93%)^&^	**Cl 172% (136%–207%)** ^**&**^ **, *t***_**1/2**_ **71% (65%–85%)** ^**&**^, *t*_1/2_ 84% (66%–100%)^&^	3rd
Darunavir [138–140]	3	85/99	19.3	NR	*C*_trough_ 87% (82%–92%), ***C***_**max**_ **73% (71%–78), AUC 75% (61%–79%)**	**Cl 150% (137%–163%),** Cl 128% (121%–136%), *t*_1/2_ 118%^&^	3rd
Didanosine [141]	1	20/20	19	*V*_d_ 119%^&^	*C*_max_ 96% (92%–101%), AUC 83%^&^	**Cl 127%,** Cl 79%, *t*_1/2_ 98% (93%–103%)	3rd
Efavirenz [142–144]	3	269/112	20	NR	***C***_**trough**_ **69% (49%–90%)** ^&^, *C*_max_ 88% (71%– 106%)^&^, **AUC 70%** ^&^, AUC 95%^&^	**Cl 123% (104%–142%)** ^**&**^	1st -3rd
Emtricitabine [145–147]	3	159/143	19.6	*V*_d_ 110%^&^	***C***_**trough**_ **68%** ^&^, *C*_trough_ 92%^&^, ***C***_**max**_ **88%** ^&^, *C*_max_ 100%^&^, **AUC 73%,** AUC 83% (82%–84%)	**Cl 121% (117%–140%),** *t*_1/2_ 96% (92%–100%)^&^	3rd
Indinavir [148–150]	3	42/47	14.6	NR	***C***_**trough**_ **36% (26%–46%)** ^&^ **, *C***_**max**_ **51% (35%–67%)** ^&^ **, AUC 59% (26%–82%)**	**Cl 215% (167%–344%)**	2nd–3rd
Lamivudine [151]	1	47/114	17	NR	NR	**Cl 122%**	2nd–3rd
Lopinavir [45,142,152–162]	13	550/454	18	***V***_**d**_ **173%** ^&^, *V*_d_ 85%^&^, **free fraction 117%** ^&^	***C***_**trough**_ **62% (34%–76%)** ^&^, *C*_trough_ 70% (68%–119%)^&^, ***C***_**max**_ **73% (54%–75%)** ^&^, *C*_max_ 83% (75%–92%)^&^, **AUC 66% (57%–74%)** ^&^, AUC 77% (71%–84%)^&^	**Cl 206% (174%–261%)** ^&^, Cl 140% (119%–147%)^&^, ***t***_**1/2**_ **63%** ^&^, *t*_1/2_ 80% (70%–106%)^&^	2nd–3rd
Nelfinavir [42,149,163–168]	8	207/191	17.2	***V***_**d**_ **71%** ^&^, *V*_d_ 106% (90%–123%)^&^	***C***_**trough**_ **52% (23%–79%)** ^&^, *C*_trough_ 75% (60%–90%)^&^, ***C***_**max**_ **73% (69%–77%)** ^&^, *C*_max_ 74% (63%–77%)^&^, **AUC 72% (61%–79%)** ^&^, AUC 69% (53%–76%)^&^	**Cl 139% (125%–153%)** ^&^, Cl 157% (100%–170%)^&^, ***t***_**1/2**_ **70% (66%–71%)** ^&^, *t*_1/2_ 76%^&^	2nd–3rd
Nevirapine [169–171]	3	192/86	19.6	NR	***C***_**trough**_ **79%** ^&^ **, *C***_**max**_ **79%** ^&^ **, AUC 79%** ^&^	NR	2nd–3rd
Raltegravir [172,173]	2	56/62	17.5	*V*_d_ 144% (138%–151%)	*C*_trough_ 92% (64%–120%), ***C***_**max**_ **58%,** *C*_max_ 81%, **AUC 46%,** AUC 70%	Cl 178% (142%–214%), *t*_1/2_ 101% (100%–102%)	3rd
Ritonavir [38,45,133–135,139,148,155–160,174–177]	17	324/394	18	***V***_**d**_ **253% (234%–273%)** ^&^, *V*_d_ 190% (121%–252%)^&^	***C***_**trough**_ **56% (42%–100%)** ^&^, *C*_trough_ 66% (34%–100%)^&^, ***C***_**max**_ **49% (32%–70%)** ^&^, *C*_max_ 58% (44%–101%)^&^, **AUC 53% (36%–71%)** ^&^, AUC 55% (35%–82%)^&^	**Cl 228% (168%–282%)** ^&^, Cl 151% (119%–206%)^&^, *t*_1/2_ 94% (60%–150%)^&^	2nd–3rd
Saquinavir [38,174–176]	4	45/69	18	*V*_d_ 91%^&^	*C*_trough_ 74% (30%–107%)^&^, ***C***_**max**_ **34%,** *C*_max_ 82% (79%–93%), **AUC 64%,** AUC 83% (43%–94%)	Cl 100% (81%–154%)^&^, *t*_1/2_ 97% (92%–112%)	2nd–3rd
Sulfadoxine [178]	1	10/28	17	*V*_d_ 113%	**AUC 56%**	**Cl 178%, *t***_**1/2**_ **57%**	2nd
Tenofovir [44,145,179,180]	4	246/155	18.5	***V***_**d**_ **128** ^&^	***C***_**trough**_ **83% (78%–88%)** ^&^, *C*_trough_ 93%^&^, ***C***_**max**_ **84%** ^&^, *C*_max_ 91% (82%–100%)^&^, **AUC 79% (77%–80%)** ^&^, AUC 80% (66%–94%)^&^	**Cl 125% (123%–127%)** ^&^ **, *t***_**1/2**_ **129%** ^&^, *t*_1/2_ 100%^&^	1st–3rd

Significant results are marked in bold.

^&^Parameter not reported in all studies.

NR, not reported.

**Table 15:** Antimalarial drugs: consistent/single studies of pregnancy-associated pharmacokinetic changes (percent calculated as pregnant/nonpregnant values).

Drug [Reference]	Number of Studies	Total Number of Women (Nonpregnant/Pregnant)	Average Quality (24 Items)	Distribution Parameters	Exposure Parameters	Elimination Parameters	Trimester
Artemeter [181,182]	2	22/46	19	NR	***C***_**max**_ **52%** ^**&**^ **, AUC 31%** ^**&**^	NR	2nd–3rd
Atovaquone [183]	1	0^!^/9	18	***V***_**d**_ **217%**	***C***_**trough**_ **22%, *C***_**max**_ **37%, AUC 21%**	**Cl 821%**	2nd–3rd
Chloroquine [184–187]	4	50/70	18.7	***V***_**d**_ **106%** ^**&**^	*C*_max_ 106% (76%–137%), **AUC 74%** ^**&**^, AUC 81% (72%–91%)^**&**^	**Cl 138% (133%–144%)** ^**&**^, Cl 110%^**&**^, ***t***_**1/2**_ **91%** ^**&**^, *t*_1/2_ 86%^**&**^	2nd–3rd
Lumefantrine [181,182,188,189]	4	56/188	19.2	*V*_d_ 90%^**&**^	**Lower concentration** ^**&**^ ^,^ ^β^, *C*_max_ 101% (100%–103%)^**&**^, AUC 97% (90%114%)^**&**^	**Higher Cl** ^**&**^ ^,^ ^β^, Cl 88%^**&**^, ***t***_**1/2**_ **81%** ^**&**^, *t*_1/2_ 151%^**&**^	2nd–3rd
Mefloquine [190–192]	3	32/53	17.6	***V***_**d**_ **108%** ^**&**^, *V*_d_ 121%^**&**^	***C***_**max**_ **77%** ^**&**^, *C*_max_ 103%^**&**^, AUC 112%	**Cl 162%,** Cl 104% (100–109%), ***t***_**1/2**_ **134%,** *t*_1/2_ 78% (68%–88%)	1st–3rd
Piperaquine [193–195]	3	81/80	19	***V***_**d**_ **66% (63%–68%),** *V*_d_ 93%	***C***_**max**_ **134%** ^**&**^, *C*_max_ 126%^**&**^, **AUC 66%,** AUC 103% (110%–117%)^**&**^	**Cl 137%,** Cl 93% (90%–96%), ***t***_**1/2**_ **72% (69%–90%)**	2nd–3rd
Proguanil [183,196]	2	4^!^/19	16.5	***V***_**d**_ **109%**	*C*_trough_ 101%^**&**^, *C*_max_ 80% (65%–95%), AUC 77% (60%–95%)	Cl 116% (73%–160%), ***t***_**1/2**_ **71%,** *t*_1/2_ 123%	2nd–3rd

Significant results are marked in bold.

^&^Parameter not reported in all studies.

^!^Data compared to published reports.

^β^Numbers were not provided.

NR, not reported.

**Table 16:** Antimalarial drugs: inconsistent studies of pregnancy-associated pharmacokinetic changes (percent calculated as pregnant/nonpregnant values).

Drug [Reference]	Number of Studies	Total Number of Women (Nonpregnant/Pregnant)	Average Quality	Distribution Parameters	Exposure Parameters	Elimination Parameters	Potential Sources for Inconsistency	Trimester
DHA (active metabolite of artesunate) [192–194,197,198]	5	169/184	18.5	***V***_**d**_ **67%,** *V*_d_ 97% (74%–108%)	**AUC 106% (82%–129%),** AUC 88% (81%–112%), *C*_max_ 113% (92%–114%)^&^	**Cl 95% (80%–110%),** Cl 106% (89%–123%), ***t***_**1/2**_ **79%,** *t*_1/2_ 84% (76%–97%)	Different disease severity, different pregnancy and nonpregnancy stages	2nd–3rd
Pyrimethamine [199,200]	2	107/127	19.5	**Inconsistent data for *V***_**d**_ ^**#**^	***C***_**max**_ **149% (142%–159%)** ^&^ **, inconsistent data for AUC** ^**#**^	**Inconsistent data for Cl** ^**#**^ **, *t***_**1/2**_ **160% (132%–189%),** *t*_1/2_ 107%	Different study designs, quality and quantity of controls and genetic variations	2nd–3rd
Sulfadoxine [199,200]	2	107/127	19.5	**Inconsistent data for *V***_**d**_ ^**#**^	***C***_**max**_ **135%** ^&^, *C*_max_ 92%^&^, **AUC 83% (67%–99),** AUC 83%	**Cl 125% (100%–151%),** Cl 125%, ***t***_**1/2**_ **80% (74%–86),** *t*_1/2_ 89%	Different study designs, quality and quantity of controls and genetic variations	2nd–3rd

Significant results are marked in bold.

^&^Parameter not reported in all studies.

^#^Number not provided.

**Table 17:** Miscellaneous classes: consistent/single studies of pregnancy associated pharmacokinetic changes (percent calculated as pregnant/nonpregnant values).

Class	Drug [Reference]	Number of Studies	Total Number of Women (Nonpregnant/Pregnant)	Average Quality (24 Items)	Distribution Parameters	Exposure Parameters	Elimination Parameters	Trimester
**Addiction management**	Buprenorphine [201]	1	3/3	20	NR	***C***_**max**_ **15%, AUC 12%**	NR	3rd
	Methadone [202–204]	3	37/56	17.3	NR	***C***_**trough**_ **30%** ^**&**^ **, AUC 25%** ^**&**^	**Cl 165% (155%–175%),** Cl 190%	2nd–3rd
**Anticancer Chemotherapy**	Carboplatin [205]	1	2/2	10	***V***_**d**_ **138%**	***C***_**max**_ **63%, AUC 58%**	**Cl 165%, *t***_**1/2**_ **82%**	2nd–3rd
	Cisplatin [206]	1	6/6	13	**Free fraction (8 h) 179%**	NR	NR	3rd
	Epirubicin [205]	1	4/4	11	***V***_**d**_ **121%**	***C***_**max**_ **60%, AUC 72%**	**Cl 142%, *t***_**1/2**_ **85%,**	2nd–3rd
	Paclitaxel [205]	1	2/5	11	***V***_**d**_ **167%**	***C***_**max**_ **54%, AUC 83%**	**Cl 120%, *t***_**1/2**_ **133%**	2nd–3rd
**Drugs for endocrine disorders**	Insulin [207]	1	10/10	15	NR	NR	**Cl 80%**	3rd
	Metformin [208–210]	3	23/69	18.3	*V*_d_ 118%^**&**^	**AUC 73%** ^**&**^	**Cl 131%** ^**&**^, *t*_1/2_ 107%^**&**^	3rd
	Thyroid releasing hormone [211]	1	8/24	17	***V***_**d**_ **146%**	***C***_**max**_ **68%, AUC 45%**	**Cl 192%, *t***_**1/2**_ **68%**	2nd–3rd
	Vasopressin [212]	1	6/6	15	NR	NR	**Higher metabolic clearance rate** ^#^	3rd
**Labor and delivery**	Ritodrine [213]	1	10/10	12	NR	*C*_max_ 80%**, AUC 72%**	NR	2nd–3rd
	Terbutaline [214]	1	3/3	10	NR	NR	**Cl 133%**	3rd
	Nifedipine [215]	1	0^!^/8	21	**Larger *V***_**d**_ ^#^	NR	**Shorter *t***_**1/2**_ ^#^	2nd–3rd

Significant results are marked in bold.

^&^Parameter not reported in all studies.

^#^Numbers were not provided.

^!^Data compared to published reports.

NR, not reported.

**Table 18:** Miscellaneous classes: inconsistent studies of pregnancy-associated pharmacokinetic changes (percent calculated as pregnant/nonpregnant values).

Class	Drug [Reference]	Number of Studies	Total Number of Women (Nonpregnant/Pregnant)	Average Quality	Distribution Parameters	Exposure Parameters	Elimination Parameters	Potential Sources for Inconsistency	Trimester
Anticancer chemotherapy	Doxorubicin [205,216]	2	5/14	15	***V***_**d**_ **129%** ^**&**^	***C***_**max**_ **66%** ^**&**^ **, AUC 75%** ^**&**^	**Inconsistent data for Cl** ^**#**^, *t*_1/2_ 101% (100%–102%)	Comparison group selection, numbers too small to draw conclusions	2nd–3rd

Significant results are marked in bold.

^&^Parameter not reported in all studies.

^#^Numbers not provided.

Table 19 shows drugs for which all the studies (36) reported no statistically significant PK differences between pregnant and nonpregnant women. Most of the drugs presented in Table 19 were only investigated in one study, while sertraline, propranolol, quinine folic acid and vitamin D3 were each presented in two publications. For sertraline, statistically non-significant decreases in the exposure parameters were reported [70,217]. In the case of propranolol, mean elimination half-life in pregnancy was shorter in both studies, but the exposure parameter (AUC) changes were not consistent; non-significant increase in the AUC [218] versus non-significant decrease in AUC [219]. Consistent but non-significant increase in Cl was reported for quinine [189,220–222]. Plasma folate concentrations showed no statistically significant changes [221,222], but conflicting change directions were seen in the mean values, depending on the dose [222]. Similarly, vitamin D3 showed conflicting change directions in exposure parameters, which were statistically non-significant [223,224].

**Table 19:** Non-significant pharmacokinetic differences between pregnant and nonpregnant women.

Class	Drug [Reference]	Number of Studies	Total Number of Women (Nonpregnant/Pregnant)	Average Quality	Distribution Parameters	Exposure Parameters	Elimination Parameters	Trimester
**Antibiotics**	Azlocillin [65]	1	4/7	9	*V*_d_ 62%	*C*_max_ 327%	Cl 64%, *t*_1/2_ 90%	2nd
	Cefotiam [225]	1	6/14	15	*V*_d_ 246%	NR	Cl 260%, *t*_1/2_ 132%	3rd
	Ceftriaxone [53]	1	4/18	18	*V*_d_ 81%	NR	Cl 50%, *t*_1/2_ 183%	3rd
	Gentamicin [53]	1	4/18	18	*V*_d_ 75%	NR	Cl 105%, *t*_1/2_ 76%	3rd
	Sulbactam [68]	1	10/10	14	*V*_d_ 87%	*C*_trough_ 96%, AUC 78%	Cl 117%, *t*_1/2_−87%	3rd
**Antidepressants**	Sertraline [70,217]	2	9/12	14	NR	*C*_max_ 54%, AUC 40%	NR	3rd
**Analgesia and anesthesia drugs**	Metamizole [226]	1	8/7	11	*V*_d_ 120%	*C*_max_ 149%, AUC 118%	Cl 73%, *t*_1/2_ 89%	3rd
	Atracurium [227]	1	8/8	17	*V*_d_ 97%	NR	Cl 105%, *t*_1/2_ 91%	NR
	Bupivacaine [228]	1	6/6	16	NR	*C*_max_140%, AUC 155%	*t*_1/2_ 111%	3rd
	Pethidine [229]	1	11/13	13	*V*_d_ 106%	*C*_max_ 51%, AUC 65%	Cl 105%, *t*_1/2_ 179%	3rd
**Cardiovascular drugs**	Alprenolol [48]	1	4/11	15	Free fraction 128%	NR	NR	3rd
	Propranolol [218,219]	2	19/19	17	*V*_d_ 70%^&^	AUC 99% (97%–101%)	Cl 106%^&^, *t*_1/2_ 79% (70%–88%)	3rd
**Antiemetics**	Pyridoxine [50]	1	18/56	19	NR	NR	Cl 108%	1st
	Doxylamine [50]	1	18/56	19	NR	NR	Cl 80%	1st
	Ondansetron [230]	1	20/40	20	NR	NR	NR	3rd
**Antiretrovirals**	Pyrimethamine [178]	1	9/28	17	*V*_d_ 93%	AUC 82%	Cl 120%, *t*_1/2_ 93%	2nd
	Zidovudine [231]	1	0^!^/8	16	NR	NR	NR	3rd
**Drugs for endocrine disorders**	Mifepristone (RU 4861) [232]	1	9/36	17	*V*_d_ 138%	*C*_max_ 83%, AUC 77%	Cl 140%	1st–3rd
	Propylthiouracil [233]	1	6/6	13	NR	AUC 52%	NR	3rd
	Thyroxine [234]	1	16/16	11	NR	No change in required dose	NR	1st–3rd
**Drugs for immune disorders**	Intravenous immunoglobulin [235]	1	5/5	19	NR	*C*_trough_ 108%, *C*_max_ 111%, AUC 102%	NR	2nd
**Antimalarial drugs**	Amodiaquine [236]	1	18/24	17	NR	*C*_max_ 102%, AUC 108%	Cl 92%, *t*_1/2_ 104%	2nd–3rd
	Quinine [189,220,237]	3	8^!^/49	19	NR	*C*_max_ 138%^&^AUC 88%^&^	Cl 120%^&^, *t*_1/2_ 110%^&^	1st–3rd
**Labor and delivery**	Atosiban [238]	1	0^!^/8	15	NR	NR	NR	2nd–3rd
	Oxytocin [239]	1	6/10	15	NR	NR	NR	3rd
	Salbutamol [240]	1	0^!^/5	14	NR	AUC 82%	Cl 104%	2nd–3rd
**Supplements**	Folic acid [221,222]	2	24/24	18	NR	Plasma folate concentrations^#^	Urine excretion^#^	2nd–3rd
	Iron [241]	1	9/10	11	*V*_d_ 70%	*C*_max_ 134%, AUC 194%	Cl 45%, *t*_1/2_ 147%	2nd–3rd
	Vitamin D3 [223,224]	2	50/55	18.5	NR	*C*_max_ 94%^&^, AUC 106%^&^	NR	3rd
**Antiviral drugs**	Acyclovir [242]	1	10/15	11	NR	*C*_trough_ 133%, *C*_max_ 100%	NR	3rd
	Oseltamivir [41]	1	23/16	15	*V*_d_ 119%	AUC 104%	Cl 92%, *t*_1/2_ 103%	1st–3rd

^&^Parameter not reported in all studies.

^!^Data compared to published reports.

^#^Numbers not provided.

NR, not reported.

Sixty of the total 218 PK observations (27.5%) reported changes in either the elimination parameters or exposure parameters. Seven PK observations (3.2%) did not report either exposure or elimination parameters. Among the 116 PK observations reporting changes in both elimination and exposure, 79.3% (92) demonstrated increased elimination together with decreased exposure in pregnant women compared to the nonpregnant population.

## Discussion

In this first systematic review, to our knowledge, of pregnancy-associated PK changes, we were able to obtain a clear overview of the landscape of the field. Now that trends of pregnancy PK change have been mapped in major drug categories and responsible metabolism or transport pathways, existing knowledge gaps critical for patient management can be addressed by the combined efforts of regulatory agencies, academia, and industry. As many women presently delay childbearing to an older age [243] and the frequency of medical conditions seen during pregnancy among older women is dramatically greater than that of younger women [244], the results of this review raise the question of whether there are sufficient data to manage these health issues appropriately during pregnancy.

Recently, the most commonly used medications in the first trimester were reported [245]. Results from 5,381 mothers identified 54 different medications used in the first trimester by at least 0.5% of pregnant women. The most commonly used prescription medications reported fell into the categories of antibiotics, analgesics, antiemetics, antidiabetic medications, and antidepressants. Among those 54 most commonly used medications, only a few had adequate data available to assess PK characteristics and dosing recommendation during pregnancy, as demonstrated by our present study results.

Although our study strived to identify all available studies describing PK changes occurring in pregnancy, the total number of these studies was relatively small. Widespread exclusion of pregnant women from clinical studies is most probably the major reason for this limitation.

Changes such as increased clearance, reduced half-life, and reduced AUC in pregnancy have been described for many drugs. These PK alterations generally lead to lower drug concentrations in plasma, decreasing maternal target exposure to drug molecules. However, whether these PK changes compromise efficacy is not necessarily certain. Indeed, the total (unbound plus bound fractions) serum concentration of a drug does not necessarily reflect its activity, as lowered plasma albumin concentration during pregnancy may increase free “active” drug concentrations, depending on the PK characteristics of the drug. Moreover, the impact of maternal dose modifications on fetal exposure requires careful planning.

Published data were inconsistent for several medications, preventing this review from defining a certain direction in PK changes. These conflicting results were seen among the antimalarial drugs (pyrimethamine [199,200], sulfadoxine [199,200], and dihydroartemisinin (DHA) [192–194,197,198]), antithrombotic drugs (unfractionated heparin [113,114] and low-molecular-weight heparin [46,114–117]), and other drugs (ampicillin [67,68] and doxorubicin [205,216]). We will discuss these drugs in detail in the following section. Also, we confirmed that the current understanding of pregnancy-associated decrease in CYP1A2 and CYP2C19 activities is not based on large studies. These findings require further validation before making clinical recommendations.

For patients who are indicated to undergo routine therapeutic drug monitoring for clinical decision making and dose titration, pregnancy may be a challenging period in which serum drug levels may decrease below the target value despite adequate adherence by patients to their regimen. As we discussed above, decrease in drug exposure levels (e.g., reduction in serum concentrations and AUC) in pregnancy may not necessarily alter clinical outcomes. The decision to change dosing schedules in patients based on therapeutic drug monitoring and/or knowledge of PK changes in pregnancy should be associated with critical assessment of the risks of therapeutic failure and adverse effects.

Fifty-one studies included in our review investigated more than one drug. Among the antiretroviral class, all studies but one presented women with HIV infection who were treated with more than one antiretroviral medication. The only study that examined a single antiretroviral drug is also the earliest study from this class, investigating zidovudine during pregnancy (published in 1993) [231]. The authors noted that in those 51 studies, no drug that interfered with absorption, elimination, distribution, etc., was included. In addition, as per Health Canada, the US Centers for Disease Control and Prevention, and the World Health Organization, antiretroviral therapy, when indicated, includes at least three agents. Therefore, it is most natural to have multiple drugs on board when conducting a PK study in HIV-positive cohorts.

### Clinical Outcome Data

The focus of the present systematic review is on PK data in pregnancy as a first step toward improving drug therapy in this orphan population. Although clinical outcomes were not reported in many of these PK studies, we identified several studies with such information.

For lamotrigine and indinavir, pregnancy-related changes in the clinical endpoints were in agreement with the observed PK changes [88,148]. Others have found significant PK changes and yet no clinical correlation was demonstrated (emtricitabine [145], levetiracetam [16], and topiramate [101]). Interestingly, while the PK-clinical correlation of some drugs was consistent among different studies (e.g., lamotrigine [86,88,91]), this was not the case for others (e.g., oxcarbazepine [96,97]). The scope of studies to investigate both PK and clinical outcome data seems to be dependent on drug class. For example, none of the studies that investigated antibiotics [47,52,53] or anesthetic and analgesic drugs [102] provided data on clinical outcomes. On the other hand, studies of addiction management drugs and antidepressant drugs reported clinical data, showing a positive correlation between decreased drug exposure and diminished clinical effects in pregnancy [70,202]. A study investigating cardiovascular drugs that reported clinical outcomes did not demonstrate significant positive clinical correlations [127]. The three drug groups that provided the richest evidence regarding clinical correlation were the antiretrovirals, antimalarials, and antiepileptics. In the case of antiretrovirals, all studies had showed decreased drug exposure in pregnancy due to PK changes. While most of these studies reported adequate viral suppression and no mother-to-child HIV transmission [132,135,138], one study reported an increased viral load during pregnancy, with a few cases of neonatal transmission of the virus [150]. Conflicting clinical results were also reported for antimalarial drugs: while some studies reported equal parasite clearance time or no increase in treatment failure in spite of decreased exposure [182], others demonstrated a positive correlation between the decreased exposure and poor clinical outcome, reporting an increase in treatment failure or a decrease in post-treatment prophylactic effect [181,195].

Our review has highlighted those medications that have relatively consistent PK change directions in pregnancy. This collection of PK data could prove to be a decision support base for future attempts to tailor medication prescription for pregnant women to achieve target serum concentrations; however, one must take into account that many studies often report undiminished drug efficacy despite the aforementioned pregnancy-associated PK changes [132,135,138,145,146,163,172,177].

### Drugs with a Consistent Pharmacokinetics Change Direction

For the vast majority of drugs (114), data gathered in this review are consistent among studies. Although not all studies presented a full set of PK parameters, the evidence exists to support the notion that in pregnancy, drug exposure levels per given dose are decreased for most medications. In addition, lower plasma protein binding (higher free drug level) is a consistent finding. This tandem trending of higher Cl rate, higher *V*_d_, and higher free fraction is observed for most drugs except for those metabolized by CYP1A2 and CYP2C19, which show a trend toward decreased metabolism during pregnancy.

### Drugs with Variable Pharmacokinetic Change Directions

Studies of seven drugs were found to yield conflicting PK results among studies in pregnancy. Three of these drugs are part of the antimalarial drug group (pyrimethamine [199,200], sulfadoxine [199,200], and DHA [192–194,197,198]), two are antithrombotic drugs (unfractionated heparin [113,114] and low-molecular-weight heparin [46,114–117]), one is an antibiotic (ampicillin [67,68]), and the last is an anticancer chemotherapeutic drug (doxorubicin [205,216]). The average quality score of the consistent antibiotic and antithrombotic studies tended to be higher than the quality score of the inconsistent studies from the same group (14.4 versus 11.5, *p* < 0.05, and 16.4 versus 15.5, *p* = 0.119, for the antibiotic and antithrombotic drugs, respectively). Nevertheless, the average quality score of the consistent studies was not higher than that of the inconsistent studies for both the antimalarial drugs (18.2 versus 19.1, *p* = 0.62) and the anticancer chemotherapeutics (11.5 versus 14.5: averages). Thus, variability of quality scores cannot account for the inconsistent PK directions that were demonstrated.

#### Ampicillin [67,68]

The pregnancy PK of ampicillin had been reported in two studies [67,68]. Both studies presented PK parameters during delivery and demonstrated conflicting results regarding the half-life of elimination. While the elimination half-life presented in one study [67] was longer among pregnant women compared to the control group (58.3 min versus 44.8 min, respectively), the other study [68] demonstrated a difference in the opposite direction (52.4 min versus 69.9 min, respectively). We believe that one of the potential sources for these conflicting results is the choice of control group: while the control group in the former [67] comprised healthy nonpregnant individuals, the post-pregnant women (who may be still under some influence of pregnancy-associated physiological changes) served as their own control in the latter study [68].

#### Pyrimethamine and sulfadoxine [199,200]

The pregnancy PK of this antimalarial drug combination had been studied in Papua New Guinea [199] and in four African countries (Mozambique, Sudan, Zambia, and Mali) [200]. These two publications present conflicting results. Concerning pyrimethamine, the Papua New Guinea time-concentration plots showed average pregnancy levels to be lower at most time points than the nonpregnant comparison, while data from the African countries indicated the opposite (measurements in pregnancy were higher). This same phenomenon was also evident in some, but not all, data reported on sulfadoxine.

Appraising the methodologies used by these two research groups, we have identified a potential source for this conflict regarding the raw data. In both studies, pregnancy was associated with significant anemia, and both papers (Table 1) reported an average reduction of ~20% in hemoglobin values during pregnancy. However, while the Papua New Guinea study used plasma for drug assays, the African study used whole blood from dried blood spots, with no correction for hematocrit values. This limitation of the dried blood spot method may have caused an overestimation of drug levels per blood spot area in pregnant women in the African study, as a result of a relative abundance of plasma per blood spot due to severe anemia [246]. Although there are likely to be other factors contributing to the discrepancies between the two studies, we speculate that the difference in the sample matrix is the major cause, and that pyrimethamine and sulfadoxine apparent clearance is higher during pregnancy. This also highlights the importance of methodological standardization in PK studies, including sample analysis procedures.

#### Dihydroartemisinin [192–194,197,198]

Five studies met the inclusion criteria that investigated the effect of pregnancy on the PK of DHA, the active metabolite of artesunate, for severe malaria (Table 16). Inconsistencies in PK parameter changes exist in the AUC and clearance of DHA; a statistically significant reduction in AUC (decreased exposure) and an increase in oral clearance in pregnancy were observed in one study [197], while the change directions were opposite in the other [198]. However, this can be explained by increased disease severity at PK sampling in the latter [198], as systemic exposure of DHA is higher in infected patients with a severe course of malaria than in those with a mild course [198,247]. The increased DHA exposure in acute malaria during pregnancy after oral artesunate is probably a result of increased bioavailability due to decreased presystemic elimination through glucuronidation in the intestine [198]. Hepatic metabolism of DHA occurs through enzymes such as CYP2B6, UGT1A9, and UGT2B7, but data on these isoenzymes in pregnant women with acute infection are still limited.

#### Low-molecular-weight heparin [46,114–117] and heparin [113,114]

Six studies investigated the PK of heparin and low-molecular-weight heparin by using factor anti-Xa activity as a surrogate marker of enoxaparin (*n* = 2), dalteparin (*n* = 3), and unfractionated heparin (*n* = 2) in pregnant women (Table 12). The statistically significant discrepancies in the pharmacokinetic parameters can be mainly attributed to the different study designs, dosing regimens, and indications for heparin in the study population (therapeutic versus prophylactic administration). However, the most important parameter in these studies is the *C*_max_ (2–4 h after administration) of the factor anti-Xa activity because it determines whether the woman is properly controlled for thromboembolic events. Studies with a dose increase design had an increase in the *C*_max_ of anti-Xa activity [114,116]. The remaining studies revealed lower *C*_max_ values during pregnancy, even with higher doses [46,113,115,117]. Those studies [46,114,117] showed higher clearance during pregnancy, which was statistically significant in two of them [46,117]. The recommended therapeutic range of 0.6–1.0 IU/ml [248] was achieved in only half of the population in one of the two studies [117]. It should be noted that the Barbour et al. [116] study compared women in the third trimester to women in early pregnancy (as the control group). Peak levels of anti-Xa activity (equivalent to *C*_max_) were 0.63 IU/ml in early pregnancy versus 0.69 IU/ml in the third trimester. These control values were somewhat higher than the *C*_max_ values reported for the other nonpregnant populations in the other studies [46,114,115].

### Study Limitations

Most studies that demonstrated significant PK changes had relatively small sample sizes. The mixture of small sample sizes with different pharmacological/research methodologies poses substantial challenges to comparing and summarizing their study results. Another limitation stems from the fact that, for many drugs, pregnancy-related PK changes were considered to be significant on the basis of a single study, often of low quality, with small numbers of women and a small subset of PK parameters. Although we show single studies with statistically significant results in the “consistent” category for simplicity of presentation, single studies do not inform on the consistency of the changes. Further replication studies are required. The quality assessment of the studies included in this review was performed using the ClinPK checklist for assessing methodological quality in clinical PK studies. This checklist provides meticulous guidelines for quality assessment, but having been only recently published, it will need refinement and external validation.

We are acutely aware of the fact that by excluding studies lacking a comparison group of nonpregnant women we may miss a significant amount of PK data. However, in the context of our research question, we find it imperative to not only document certain kinetic patterns but also provide quantitative or semiquantitative estimates of the extent and directionality of those pregnancy-associated PK changes. Comparing cohort data for pregnant women to normal population averages would expose our study to a multitude of biases, mainly due to the fact that the most dominant contributors to the “normal population” PK parameter values, in textbooks and seminal papers, are healthy men (Lexicomp and Micromedex databases, for example, report “adult” data with no gender, yet the citation lists are rich with male volunteer publications). Moreover, in the majority of studies included in this systematic review, pregnant women served as their own controls (in the prepregnancy or postpartum state), which isolates the pregnancy as the most dominant factor in the assessment.

Lastly, trimester-specific PK changes were difficult to summarize. While most of the studies provided third trimester results, others reported separate results from the second and third trimesters, and few reported separate results from all trimesters. Physiological changes in pregnancy take place progressively during gestation (reviewed by Costantine [8] and Loebstein et al. [9]). As such, we hypothesized that this would lead to trimester-specific differences in drug disposition. Unfortunately, however, many studies in this review did not report trimester-specific changes, which could possibly have contributed to the conflicting PK results in some studies described above.

## Conclusions

Our systematic analyses confirmed that many drugs are subject to pregnancy-associated PK changes, which may alter plasma/serum drug concentration profiles. However, we have also found a paucity of clinically useful data on whether dose adjustment is necessary for these PK changes. Where such PK studies were done, generally only a few PK parameters were estimated, sample sizes were small, and maternal and/or fetal outcomes were not examined. Further studies that address these limitations are needed to optimize drug therapy for pregnant women.

## Supporting Information

S1 Checklist PRISMA checklist for reporting systematic reviews. (DOC)

S1 Table Search strategy. (DOCX)

S2 Table Updated search strategy. (DOCX)

S3 Table Non-included full text studies with their reasons. (DOCX)

S4 Table Extracted data. (XLSX)

## Abbreviations

AUC: area under the curve
C/D ratio: concentration-to-dose ratio
DHA: dihydroartemisinin
OTC: over-the-counter
PK: pharmacokinetics
UGT: uridine 5′-diphospho-glucuronosyltransferase

## References

[1] Andrade SE, Gurwitz JH, Davis RL, Chan KA, Finkelstein JA, Fortman K, et al Prescription drug use in pregnancy. Am J Obstet Gynecol. 2004;191(2):398–407. 10.1016/j.ajog.2004.04.025 15343213

[2] Glover DD, Amonkar M, Rybeck BF, Tracy TS. Prescription, over-the-counter, and herbal medicine use in a rural, obstetric population. Am J Obstet Gynecol. 2003;188(4):1039–45. 12712107 10.1067/mob.2003.223

[3] Mitchell AA, Gilboa SM, Werler MM, Kelley KE, Louik C, Hernandez-Diaz S, et al Medication use during pregnancy, with particular focus on prescription drugs: 1976–2008. Am J Obstet Gynecol. 2011;205(1):51 e1–8. 21514558 10.1016/j.ajog.2011.02.029 PMC3793635

[4] Werler MM, Mitchell AA, Hernandez-Diaz S, Honein MA. Use of over-the-counter medications during pregnancy. Am J Obstet Gynecol. 2005;193(3 Pt 1):771–7. 16150273 10.1016/j.ajog.2005.02.100

[5] Bakker MK, Jentink J, Vroom F, Van Den Berg PB, De Walle HE, De Jong-Van Den Berg LT. Drug prescription patterns before, during and after pregnancy for chronic, occasional and pregnancy-related drugs in the Netherlands. BJOG. 2006;113(5):559–68. 10.1111/j.1471-0528.2006.00927.x 16637899

[6] Gagne JJ, Maio V, Berghella V, Louis DZ, Gonnella JS. Prescription drug use during pregnancy: a population-based study in Regione Emilia-Romagna, Italy. Eur J Clin Pharmacol. 2008;64(11):1125–32. 10.1007/s00228-008-0546-y 18685836

[7] Olesen C, Steffensen FH, Nielsen GL, de Jong-van den Berg L, Olsen J, Sorensen HT. Drug use in first pregnancy and lactation: a population-based survey among Danish women. The EUROMAP group. Eur J Clin Pharmacol. 1999;55(2):139–44. 10335909 10.1007/s002280050608

[8] Costantine MM. Physiologic and pharmacokinetic changes in pregnancy. Front Pharmacol. 2014;5:65 10.3389/fphar.2014.00065 24772083 PMC3982119

[9] Loebstein R, Lalkin A, Koren G. Pharmacokinetic changes during pregnancy and their clinical relevance. Clin Pharmacokinet. 1997;33(5):328–43. 10.2165/00003088-199733050-00002 9391746

[10] Anderson GD. Pregnancy-induced changes in pharmacokinetics: a mechanistic-based approach. Clin Pharmacokinet. 2005;44(10):989–1008. 10.2165/00003088-200544100-00001 16176115

[11] Anderson GD. Using pharmacokinetics to predict the effects of pregnancy and maternal-infant transfer of drugs during lactation. Expert Opin Drug Metab Toxicol. 2006;2(6):947–60. 10.1517/17425255.2.6.947 17125410

[12] DeVane CL, Stowe ZN, Donovan JL, Newport DJ, Pennell PB, Ritchie JC, et al Therapeutic drug monitoring of psychoactive drugs during pregnancy in the genomic era: challenges and opportunities. J Psychopharmacol. 2006;20(4 Suppl):54–9. 10.1177/1359786806066054 16785271

[13] Leppik IE, Rask CA. Pharmacokinetics of antiepileptic drugs during pregnancy. Semin Neurol. 1988;8(3):240–6. 10.1055/s-2008-1041385 3057557

[14] McAuley JW, Anderson GD. Treatment of epilepsy in women of reproductive age: pharmacokinetic considerations. Clin Pharmacokinet. 2002;41(8):559–79. 10.2165/00003088-200241080-00002 12102641

[15] Pennell PB. Antiepileptic drug pharmacokinetics during pregnancy and lactation. Neurology. 2003;61(6 Suppl 2):S35–42. 10.1212/wnl.61.6_suppl_2.s35 14504308

[16] Tomson T, Palm R, Kallen K, Ben-Menachem E, Soderfeldt B, Danielsson B, et al Pharmacokinetics of levetiracetam during pregnancy, delivery, in the neonatal period, and lactation. Epilepsia. 2007;48(6):1111–6. 10.1111/j.1528-1167.2007.01032.x 17381438

[17] Wadelius M, Darj E, Frenne G, Rane A. Induction of CYP2D6 in pregnancy. Clin Pharmacol Ther. 1997;62(4):400–7. 10.1016/S0009-9236(97)90118-1 9357391

[18] Tsutsumi K, Kotegawa T, Matsuki S, Tanaka Y, Ishii Y, Kodama Y, et al The effect of pregnancy on cytochrome P4501A2, xanthine oxidase, and N-acetyltransferase activities in humans. Clin Pharmacol Ther. 2001;70(2):121–5. 10.1067/mcp.2001.116495 11503005

[19] Koren G. Pharmacokinetics in pregnancy; clinical significance. J Popul Ther Clin Pharmacol. 2011;18(3):e523–7. 22113390

[20] Anger GJ, Piquette-Miller M. Pharmacokinetic studies in pregnant women. Clin Pharmacol Ther. 2008;83(1):184–7. 10.1038/sj.clpt.6100377 17882157

[21] Dickmann LJ, Isoherranen N. Quantitative prediction of CYP2B6 induction by estradiol during pregnancy: potential explanation for increased methadone clearance during pregnancy. Drug Metab Dispos. 2013;41(2):270–4. 10.1124/dmd.112.047118 22815312

[22] Choi SY, Koh KH, Jeong H. Isoform-specific regulation of cytochromes P450 expression by estradiol and progesterone. Drug Metab Dispos. 2013;41(2):263–9. 10.1124/dmd.112.046276 22837389 PMC3558868

[23] Ke AB, Nallani SC, Zhao P, Rostami-Hodjegan A, Unadkat JD. Expansion of a PBPK model to predict disposition in pregnant women of drugs cleared via multiple CYP enzymes, including CYP2B6, CYP2C9 and CYP2C19. Br J Clin Pharmacol. 2014;77(3):554–70. 10.1111/bcp.12207 23834474 PMC4371535

[24] Tomson T, Lindbom U, Ekqvist B, Sundqvist A. Disposition of carbamazepine and phenytoin in pregnancy. Epilepsia. 1994;35(1):131–5. 8112235 10.1111/j.1528-1157.1994.tb02922.x

[25] McGready R, Stepniewska K, Seaton E, Cho T, Cho D, Ginsberg A, et al Pregnancy and use of oral contraceptives reduces the biotransformation of proguanil to cycloguanil. Eur J Clin Pharmacol. 2003;59(7):553–7. 10.1007/s00228-003-0651-x 12955370

[26] Lee JK, Chung HJ, Fischer L, Fischer J, Gonzalez FJ, Jeong H. Human placental lactogen induces CYP2E1 expression via PI 3-kinase pathway in female human hepatocytes. Drug Metab Dispos. 2014;42(4):492–9. 10.1124/dmd.113.055384 24408518 PMC3965907

[27] Aweeka FT, Hu C, Huang L, Best BM, Stek A, Lizak P, et al Alteration in cytochrome P450 3A4 activity as measured by a urine cortisol assay in HIV-1-infected pregnant women and relationship to antiretroviral pharmacokinetics. HIV Med. 2015;16(3):176–83. 10.1111/hiv.12195 25407158 PMC4320673

[28] Chen H, Yang K, Choi S, Fischer JH, Jeong H. Up-regulation of UDP-glucuronosyltransferase (UGT) 1A4 by 17beta-estradiol: a potential mechanism of increased lamotrigine elimination in pregnancy. Drug Metab Dispos. 2009;37(9):1841–7. 10.1124/dmd.109.026609 19546240 PMC2729326

[29] Roustit M, Jlaiel M, Leclercq P, Stanke-Labesque F. Pharmacokinetics and therapeutic drug monitoring of antiretrovirals in pregnant women. Br J Clin Pharmacol. 2008;66(2):179–95. 10.1111/j.1365-2125.2008.03220.x 18537960 PMC2492933

[30] Pennell PB. Antiepileptic drugs during pregnancy: what is known and which AEDs seem to be safest? Epilepsia. 2008;49(Suppl 9):43–55. 10.1111/j.1528-1167.2008.01926.x 19087117 PMC3882069

[31] Anderson GD, Carr DB. Effect of pregnancy on the pharmacokinetics of antihypertensive drugs. Clin Pharmacokinet. 2009;48(3):159–68. 10.2165/00003088-200948030-00002 19385709

[32] Clark CT, Klein AM, Perel JM, Helsel J, Wisner KL. Lamotrigine dosing for pregnant patients with bipolar disorder. Am J Psychiatry. 2013;170(11):1240–7. 10.1176/appi.ajp.2013.13010006 24185239 PMC4154145

[33] Klieger C, Pollex E, Kazmin A, Koren G. Hypoglycemics: pharmacokinetic considerations during pregnancy. Ther Drug Monit. 2009;31(5):533–41. 10.1097/FTD.0b013e3181b385ba 19730277

[34] Deligiannidis KM, Byatt N, Freeman MP. Pharmacotherapy for mood disorders in pregnancy: a review of pharmacokinetic changes and clinical recommendations for therapeutic drug monitoring. J Clin Psychopharmacol. 2014;34(2):244–55. 10.1097/JCP.0000000000000087 24525634 PMC4105343

[35] McCormack SA, Best BM. Obstetric pharmacokinetic dosing studies are urgently needed. Front Pediatr. 2014;2:9 10.3389/fped.2014.00009 24575394 PMC3920104

[36] Moher D, Liberati A, Tetzlaff J, Altman DG, Group P. Preferred reporting items for systematic reviews and meta-analyses: the PRISMA statement. PLoS Med. 2009;6(7):e1000097 10.1371/journal.pmed.1000097 19621072 PMC2707599

[37] Kanji S, Hayes M, Ling A, Shamseer L, Chant C, Edwards DJ, et al Reporting guidelines for clinical pharmacokinetic studies: the ClinPK Statement. Clin Pharmacokinet. 2015;54(7):783–95. 10.1007/s40262-015-0236-8 25637173

[38] Acosta EP, Bardeguez A, Zorrilla CD, Van Dyke R, Hughes MD, Huang S, et al Pharmacokinetics of saquinavir plus low-dose ritonavir in human immunodeficiency virus-infected pregnant women? Antimicrob Agents Chemother. 2004;48(2):430–6. 10.1128/AAC.48.2.430-436.2004 14742191 PMC321538

[39] Allegaert K, Van Mieghem T, Verbesselt R, Vanhole C, Devlieger R, Cossey V, et al Cefazolin plasma protein binding saturability during pregnancy. Methods Find Exp Clin Pharmacol. 2009;31(1):25–8. 10.1358/mf.2009.31.1.1338413 19357795

[40] Aquirre C, Rodriguez-Sasiain JM, Navajas P, Calvo R. Plasma protein binding of penbutolol in pregnancy. Eur J Drug Metab Pharmacokinet. 1988;13(1):23–6. 3396610 10.1007/BF03189924

[41] Beigi RH, Han K, Venkataramanan R, Hankins GD, Clark S, Hebert MF, et al Pharmacokinetics of oseltamivir among pregnant and nonpregnant women. Am J Obstet Gynecol. 2011;204(6 Suppl):S84–8. 10.1016/j.ajog.2011.03.002 21492826 PMC3111757

[42] Bryson YJ, Mirochnick M, Stek A, Mofenson LM, Connor J, Capparelli E, et al Pharmacokinetics and safety of nelfinavir when used in combination with zidovudine and lamivudine in HIV-infected pregnant women: Pediatric AIDS Clinical Trials Group (PACTG) Protocol 353. HIV Clin Trials. 2008;9(2):115–25. 10.1310/hct0902-115 18474496 PMC4043374

[43] Andrew MA, Easterling TR, Carr DB, Shen D, Buchanan ML, Rutherford T, et al Amoxicillin pharmacokinetics in pregnant women: Modeling and simulations of dosage strategies. Clin Pharmacol Ther. 2007;81(4):547–56. 10.1038/sj.clpt.6100126 17329990

[44] Best BM, Burchett S, Li H, Stek A, Hu C, Wang J, et al Pharmacokinetics of tenofovir during pregnancy and postpartum. HIV Med. 2015;16(8):502–11. 10.1111/hiv.12252 25959631 PMC4862736

[45] Best BM, Stek AM, Mirochnick M, Hu C, Li H, Burchett SK, et al Lopinavir tablet pharmacokinetics with an increased dose during pregnancy. J Acquire Immune Defic Syndr. 2010;54(4):381–8. 10.1097/qai.0b013e3181d6c9ed PMC3265163 20632458

[46] Casele HL, Laifer SA, Woelkers DA, Venkataramanan R. Changes in the pharmacokinetics of the low-molecular-weight heparin enoxaparin sodium during pregnancy. Am J Obstet Gynecol. 1999;181(5 Pt 1):1113–7. 10561628 10.1016/s0002-9378(99)70091-8

[47] Fischer JH, Sarto GE, Habibi M, Kilpatrick SJ, Tuomala RE, Shier JM, et al Influence of body weight, ethnicity, oral contraceptives, and pregnancy on the pharmacokinetics of azithromycin in women of childbearing age. Antimicrob Agents Chemother. 2012;56(2):715–24. 10.1128/AAC.00717-11 22106226 PMC3264225

[48] Herngren L, Ehrnebo M, Boreus LO. Drug binding to plasma proteins during human pregnancy and in the perinatal period. Studies on cloxacillin and alprenolol. Dev Pharmacol Ther. 1983;6(2):110–24. 6861596 10.1159/000457284

[49] Beaulac-Baillargeon L, Rocheleau S. Paracetamol pharmacokinetics during the first trimester. Eur J Clin Pharmacol. 1994;46(5):451–4. 7957542 10.1007/BF00191910

[50] Matok I, Clark S, Caritis S, Miodovnik M, Umans J, Hankins G, et al Comparing the pharmacokinetics of doxylamine/pyridoxine delayed-release combination in nonpregnant women of reproductive age and women in the first trimester of pregnancy. J Clin Pharmacol. 2013;53(3):334–8. 10.1177/0091270012445207 23444286

[51] Salman S, Rogerson SJ, Kose K, Griffin S, Gomorai S, Baiwog F, et al Pharmacokinetic properties of azithromycin in pregnancy. Antimicrob Agents Chemother. 2010;54(1):360–6. 10.1128/AAC.00771-09 19858250 PMC2798488

[52] Papantoniou N, Ismailos G, Daskalakis G, Karabinas C, Mesogitis S, Papapanagiotou A, et al Pharmacokinetics of oral cefatrizine in pregnant and non-pregnant women with reference to fetal distribution. Fetal Diagn Ther. 2007;22(2):100–6. 10.1159/000097105 17135753

[53] Popovic J, Grujic Z, Sabo A. Influence of pregnancy on ceftriaxone, cefazolin and gentamicin pharmacokinetics in caesarean vs. non-pregnant sectioned women. J Clin Pharm Ther. 2007;32(6):595–602. 10.1111/j.1365-2710.2007.00864.x 18021337

[54] Philipson A, Stiernstedt G, Ehrnebo M. Comparison of the pharmacokinetics of cephradine and cefazolin in pregnant and non-pregnant women. Clin Pharmacokinet. 1987;12(2):136–44. 10.2165/00003088-198712020-00004 3829560

[55] Gonik B, Feldman S, Pickering LK, Doughtie CG. Pharmacokinetics of cefoperazone in the parturient. Antimicrob Agents Chemother. 1986;30(6):874–6. 3813513 10.1128/aac.30.6.874 PMC180610

[56] Nathorst-Boos J, Philipson A, Hedman A, Arvisson A. Renal elimination of ceftazidime during pregnancy. Am J Obstet Gynecol. 1995;172(1):163–6. 7847529 10.1016/0002-9378(95)90107-8

[57] Philipson A, Stiernstedt G. Pharmacokinetics of cefuroxime in pregnancy. Am J Obstet Gynecol. 1982;142(7):823–8. 7065060 10.1016/s0002-9378(16)32526-1

[58] Herngren L, Ehrnebo M, Boreus LO. Drug distribution in whole blood of mothers and their newborn infants: studies of cloxacillin and flucloxacillin. Eur J Clin Pharmacol. 1982;22(4):351–8. 7106171 10.1007/BF00548405

[59] Heikkila A, Renkonen OV, Erkkola R. Pharmacokinetics and transplacental passage of imipenem during pregnancy. Antimicrob Agents Chemother. 1992;36(12):2652–5. 1482132 10.1128/aac.36.12.2652 PMC245522

[60] Heikkila A, Pyykko K, Erkkola R, Iisalo E. The pharmacokinetics of mecillinam and pivmecillinam in pregnant and non-pregnant women. Br J Clin Pharmacol. 1992;33(6):629–33. 1389936 10.1111/j.1365-2125.1992.tb04092.x PMC1381355

[61] Nemutlu E, Kir S, Eroglu H, Katlan D, Ozek A, Ozyuncu O, et al Comparison of pharmacokinetic profiles of moxifloxacin in Caesarean versus non-pregnant sectioned women by fully validated HPLC with fluorescence detection. Comb Chem High Throughput Screen. 2010;13(6):502–9. 20426751 10.2174/138620710791516003

[62] Heikkila AM, Erkkola RU. The need for adjustment of dosage regimen of penicillin V during pregnancy. Obstet Gynecol. 1993;81(6):919–21. 8497356

[63] Heikkila A, Erkkola R. Pharmacokinetics of piperacillin during pregnancy. J Antimicrob Chemother. 1991;28(3):419–23. 1960122 10.1093/jac/28.3.419

[64] Bourget P, Sertin A, Lesne-Hulin A, Fernandez H, Ville Y, Van Peborgh P. Influence of pregnancy on the pharmacokinetic behaviour and the transplacental transfer of the piperacillin-tazobactam combination. Eur J Obstet Gynecol Reprod Biol. 1998;76(1):21–7. 9481541 10.1016/s0301-2115(97)00150-4

[65] Voigt R, Schroder S, Peiker G. Pharmacokinetic studies of azlocillin and piperacillin during late pregnancy. Chemotherapy. 1985;31(6):417–24. 3908007 10.1159/000238369

[66] Peiker G, Traeger A, Pischke U, Schroder S, Muller B, Noschel H. [Studies on the pharmacokinetics of the compound preparation sulfamerazine/trimethoprim (Berlocombin-200) in pregnancy]. Pharmazie. 1982;37(8):578–83. 7146066

[67] Voigt R, Schroder S, Meinhold P, Zenner I, Noschel H. [Clinical studies on the effect of pregnancy and labor on the pharmacokinetics of ampicillin]. Zentralbl Gynakol. 1978;100(11):701–5. 356490

[68] Chamberlain A, White S, Bawdon R, Thomas S, Larsen B. Pharmacokinetics of ampicillin and sulbactam in pregnancy. Am J Obstet Gynecol. 1993;168(2):667–73. 8438948 10.1016/0002-9378(93)90515-k

[69] Heikkinen T, Ekblad U, Kero P, Ekblad S, Laine K. Citalopram in pregnancy and lactation. Clin Pharmacol Ther. 2002;72(2):184–91. 10.1067/mcp.2002.126181 12189365

[70] Sit DK, Perel JM, Helsel JC, Wisner KL. Changes in antidepressant metabolism and dosing across pregnancy and early postpartum. J Clin Psychiatry. 2008;69(4):652–8. 18426260 10.4088/jcp.v69n0419 PMC2408825

[71] Heikkinen T, Ekblad U, Palo P, Laine K. Pharmacokinetics of fluoxetine and norfluoxetine in pregnancy and lactation. Clin Pharmacol Ther. 2003;73(4):330–7. 12709723 10.1016/s0009-9236(02)17634-x

[72] Brogtrop J, Zwarts P, Holleboom CAG, Van Der Linden PD, Touw DJ. [Optimisation of pharmacotherapy for depression during pregnancy. Pilot study for evaluation of existing paroxetine therapy]. Pharmaceutisch Weekblad. 2007;142(11):34–7.

[73] Ter Horst PGJ, Larmene-Beld KHM, Bosman J, Van Der Veen EL, Wieringa A, Smit JP. Concentrations of venlafaxine and its main metabolite O-desmethylvenlafaxine during pregnancy. J Clin Pharm Ther. 2014;39(5):541–4. 10.1111/jcpt.12188 24989434

[74] Rey E, d’Athis P, Giraux P, de Lauture D, Turquais JM, Chavinie J, et al Pharmacokinetics of clorazepate in pregnant and non-pregnant women. Eur J Clin Pharmacol. 1979;15(3):175–80. 37089 10.1007/BF00563102

[75] Hebert MF, Easterling TR, Kirby B, Carr DB, Buchanan ML, Rutherford T, et al Effects of pregnancy on CYP3A and P-glycoprotein activities as measured by disposition of midazolam and digoxin: a University of Washington specialized center of research study. Clin Pharmacol Ther. 2008;84(2):248–53. 10.1038/clpt.2008.1 18288078

[76] Wilson CM, Dundee JW, Moore J, Howard PJ, Collier PS. A comparison of the early pharmacokinetics of midazolam in pregnant and nonpregnant women. Anaesthesia. 1987;42(10):1057–62. 3688386 10.1111/j.1365-2044.1987.tb05168.x

[77] Battino D, Binelli S, Bossi L, Canger R, Croci D, Cusi C, et al Plasma concentrations of carbamazepine and carbamazepine 10,11-epoxide during pregnancy and after delivery. Clin Pharmacokinet. 1985;10(3):279–84. 10.2165/00003088-198510030-00007 4017398

[78] Bernus I, Hooper WD, Dickinson RG, Eadie MJ. Metabolism of carbamazepine and co-administered anticonvulsants during pregnancy. Epilepsy Res. 1995;21(1):65–75. 7641678 10.1016/0920-1211(95)00012-y

[79] Johnson EL, Stowe ZN, Ritchie JC, Newport DJ, Newman ML, Knight B, et al Carbamazepine clearance and seizure stability during pregnancy. Epilepsy Behav. 2014;33:49–53. 10.1016/j.yebeh.2014.02.011 24632353 PMC4040964

[80] Yerby MS, Friel PN, Miller DQ. Carbamazepine protein binding and disposition in pregnancy. Ther Drug Monit. 1985;7(3):269–73. 4049462 10.1097/00007691-198507030-00005

[81] Yerby MS, Friel PN, McCormick K, Koerner M, Van Allen M, Leavitt AM, et al Pharmacokinetics of anticonvulsants in pregnancy: alterations in plasma protein binding. Epilepsy Res. 1990;5(3):223–8. 2384078 10.1016/0920-1211(90)90042-t

[82] Tomson T, Lindbom U, Ekqvist B, Sundqvist A. Epilepsy and pregnancy: a prospective study of seizure control in relation to free and total plasma concentrations of carbamazepine and phenytoin. Epilepsia. 1994;35(1):122–30. 8112234 10.1111/j.1528-1157.1994.tb02921.x

[83] Reisinger TL, Newman M, Loring DW, Pennell PB, Meador KJ. Antiepileptic drug clearance and seizure frequency during pregnancy in women with epilepsy. Epilepsy Behav. 2013;29(1):13–8. 10.1016/j.yebeh.2013.06.026 23911354 PMC3775962

[84] Bernus I, Hooper WD, Dickinson RG, Eadie MJ. Effects of pregnancy on various pathways of human antiepileptic drug metabolism. Clin Neuropharmacol. 1997;20(1):13–21. 9037569 10.1097/00002826-199702000-00002

[85] Kuhnz W, Steldinger R, Nau H. Protein binding of carbamazepine and its epoxide in maternal and fetal plasma at delivery: comparison to other anticonvulsants. Dev Pharmacol Ther. 1984;7(1):61–72. 6697871 10.1159/000457144

[86] Pennell PB, Newport DJ, Stowe ZN, Helmers SL, Montgomery JQ, Henry TR. The impact of pregnancy and childbirth on the metabolism of lamotrigine. Neurology. 2004;62(2):292–5. 14745072 10.1212/01.wnl.0000103286.47129.f8

[87] Pennell PB, Peng L, Newport DJ, Ritchie JC, Koganti A, Holley DK, et al Lamotrigine in pregnancy: clearance, therapeutic drug monitoring, and seizure frequency. Neurology. 2008;70(22 Pt 2):2130–6. 18046009 10.1212/01.wnl.0000289511.20864.2a PMC3589527

[88] de Haan GJ, Edelbroek P, Segers J, Engelsman M, Lindhout D, Devile-Notschaele M, et al Gestation-induced changes in lamotrigine pharmacokinetics: a monotherapy study. Neurology. 2004;63(3):571–3. 15304599 10.1212/01.wnl.0000133213.10244.fd

[89] Fotopoulou C, Kretz R, Bauer S, Schefold JC, Schmitz B, Dudenhausen JW, et al Prospectively assessed changes in lamotrigine-concentration in women with epilepsy during pregnancy, lactation and the neonatal period. Epilepsy Res. 2009;85(1):60–4. 10.1016/j.eplepsyres.2009.02.011 19272754

[90] Ohman I, Beck O, Vitols S, Tomson T. Plasma concentrations of lamotrigine and its 2-N-glucuronide metabolite during pregnancy in women with epilepsy. Epilepsia. 2008;49(6):1075–80. 10.1111/j.1528-1167.2007.01471.x 18076642

[91] Petrenaite V, Sabers A, Hansen-Schwartz J. Individual changes in lamotrigine plasma concentrations during pregnancy. Epilepsy Res. 2005;65(3):185–8. 10.1016/j.eplepsyres.2005.06.004 16084694

[92] Polepally AR, Pennell PB, Brundage RC, Stowe ZN, Newport DJ, Viguera AC, et al Model-based lamotrigine clearance changes during pregnancy: clinical implication. Ann Clin Transl Neurol. 2014;1(2):99–106. 10.1002/acn3.29 24883336 PMC4038031

[93] Tran TA, Leppik IE, Blesi K, Sathanandan ST, Remmel R. Lamotrigine clearance during pregnancy. Neurology. 2002;59(2):251–5. 12136066 10.1212/wnl.59.2.251

[94] Lopez-Fraile IP, Cid AO, Juste AO, Modrego PJ. Levetiracetam plasma level monitoring during pregnancy, delivery, and postpartum: clinical and outcome implications. Epilepsy Behav. 2009;15(3):372–5. 10.1016/j.yebeh.2009.04.006 19362602

[95] Westin AA, Reimers A, Helde G, Nakken KO, Brodtkorb E. Serum concentration/dose ratio of levetiracetam before, during and after pregnancy. Seizure. 2008;17(2):192–8. 10.1016/j.seizure.2007.11.027 18180176

[96] Christensen J, Sabers A, Sidenius P. Oxcarbazepine concentrations during pregnancy: a retrospective study in patients with epilepsy. Neurology. 2006;67(8):1497–9. 10.1212/01.wnl.0000240047.11166.0e 17060586

[97] Petrenaite V, Sabers A, Hansen-Schwartz J. Seizure deterioration in women treated with oxcarbazepine during pregnancy. Epilepsy Res. 2009;84(2–3):245–9. 10.1016/j.eplepsyres.2009.01.011 19231139

[98] Mazzucchelli I, Onat FY, Ozkara C, Atakli D, Specchio LM, Neve AL, et al Changes in the disposition of oxcarbazepine and its metabolites during pregnancy and the puerperium. Epilepsia. 2006;47(3):504–9. 10.1111/j.1528-1167.2006.00459.x 16529613

[99] Appleton MP, Kuehl TJ, Raebel MA, Adams HR, Knight AB, Gold WR. Magnesium sulfate versus phenytoin for seizure prophylaxis in pregnancy-induced hypertension. Am J Obstet Gynecol. 1991;165(4 Pt 1):907–13. 1951552 10.1016/0002-9378(91)90437-v

[100] Ohman I, Sabers A, de Flon P, Luef G, Tomson T. Pharmacokinetics of topiramate during pregnancy. Epilepsy Research. 2009;87(2–3):124–9. 10.1016/j.eplepsyres.2009.08.004 19740626

[101] Westin AA, Nakken KO, Johannessen SI, Reimers A, Lillestolen KM, Brodtkorb E. Serum concentration/dose ratio of topiramate during pregnancy. Epilepsia. 2009;50(3):480–5. 10.1111/j.1528-1167.2008.01776.x 19178558

[102] Kulo A, van Calsteren K, Verbesselt R, Smits A, Devlieger R, de Hoon J, et al The impact of caesarean delivery on paracetamol and ketorolac pharmacokinetics: a paired analysis. J Biomed Biotechnol. 2012;2012:437639 10.1155/2012/437639 22675252 PMC3363964

[103] Gerdin E, Salmonson T, Lindberg B, Rane A. Maternal kinetics of morphine during labour. J Perinat Med. 1990;18(6):479–87. 2097341 10.1515/jpme.1990.18.6.479

[104] Rayburn W, Shukla U, Stetson P, Piehl E. Acetaminophen pharmacokinetics: comparison between pregnant and nonpregnant women. Am J Obstet Gynecol. 1986;155(6):1353–6. 3789044 10.1016/0002-9378(86)90173-0

[105] Miners JO, Robson RA, Birkett DJ. Paracetamol metabolism in pregnancy. Br J Clin Pharmacol. 1986;22(3):359–62. 3768250 10.1111/j.1365-2125.1986.tb02901.x PMC1401133

[106] Kulo A, Peeters MY, Allegaert K, Smits A, de Hoon J, Verbesselt R, et al Pharmacokinetics of paracetamol and its metabolites in women at delivery and post-partum. Br J Clin Pharmacol. 2013;75(3):850–60. 10.1111/j.1365-2125.2012.04402.x 22845052 PMC3575952

[107] Allegaert K, Peeters MY, Beleyn B, Smits A, Kulo A, van Calsteren K, et al Paracetamol pharmacokinetics and metabolism in young women. BMC Anesthesiol. 2015;15:163 10.1186/s12871-015-0144-3 26566962 PMC4644344

[108] Gin T, Yau G, Jong W, Tan P, Leung RK, Chan K. Disposition of propofol at caesarean section and in the postpartum period. Br J Anaesth. 1991;67(1):49–53. 1859759 10.1093/bja/67.1.49

[109] Gin T, Gregory MA, Chan K, Buckley T, Oh TE. Pharmacokinetics of propofol in women undergoing elective caesarean section. Br J Anaesth. 1990;64(2):148–53. 2138487 10.1093/bja/64.2.148

[110] Cortambert F, Marti-Flich J, Dasset MP, Mullet C. [The pharmacokinetics of propofol used in cesarean section; a preliminary study in the newborn infant]. Cah Anesthesiol. 1989;37(1):33–7. 2784338

[111] Chiba K, Ishizaki T, Tabuchi T, Wagatsuma T, Nakazawa Y. Antipyrine disposition in relation to lowered anticonvulsant plasma level during pregnancy. Obstet Gynecol. 1982;60(5):620–6. 6815599

[112] Rymark P, Berntorp E, Nordsjo P, Liedholm H, Melander A, Gennser G. Low-dose aspirin to pregnant women: single dose pharmacokinetics and influence of short term treatment on bleeding time. J Perinat Med. 1994;22(3):205–11. 7823260 10.1515/jpme.1994.22.3.205

[113] Brancazio LR, Roperti KA, Stierer R, Laifer SA. Pharmacokinetics and pharmacodynamics of subcutaneous heparin during the early third trimester of pregnancy. Am J Obstet Gynecol. 1995;173(4):1240–5. 7485329 10.1016/0002-9378(95)91362-9

[114] Ensom MHH, Stephenson MD. Pharmacokinetics of low molecular weight heparin and unfractionated heparin in pregnancy. J Soc Gynecol Investig. 2004;11(6):377–83. 10.1016/j.jsgi.2004.02.007 15350250

[115] Sephton V, Farquharson RG, Topping J, Quenby SM, Cowan C, Back DJ, et al A longitudinal study of maternal dose response to low molecular weight heparin in pregnancy. Obstet Gynecol. 2003;101(6):1307–11. 12798541 10.1016/s0029-7844(03)00340-5

[116] Barbour LA, Oja JL, Schultz LK. A prospective trial that demonstrates that dalteparin requirements increase in pregnancy to maintain therapeutic levels of anticoagulation. Am J Obstet Gynecol. 2004;191(3):1024–9. 10.1016/j.ajog.2004.05.050 15467584

[117] Lebaudy C, Hulot JS, Amoura Z, Costedoat-Chalumeau N, Serreau R, Ankri A, et al Changes in enoxaparin pharmacokinetics during pregnancy and implications for antithrombotic therapeutic strategy. Clin Pharmacol Ther. 2008;84(3):370–7. 10.1038/clpt.2008.73 18431408

[118] Hebert MF, Carr DB, Anderson GD, Blough D, Green GE, Brateng DA, et al Pharmacokinetics and pharmacodynamics of atenolol during pregnancy and postpartum. J Clin Pharmacol. 2005;45(1):25–33. 10.1177/0091270004269704 15601802

[119] Hurst AK, Shotan A, Hoffman K, Johnson J, Goodwin TM, Koda R, et al Pharmacokinetic and pharmacodynamic evaluation of atenolol during and after pregnancy. Pharmacotherapy. 1998;18(4):840–6. 9692658

[120] Buchanan ML, Easterling TR, Carr DB, Shen DD, Risler LJ, Nelson WL, et al Short communication clonidine pharmacokinetics in pregnancy. Drug Metab Dispos. 2009;37(4):702–5. 10.1124/dmd.108.024984 19116263 PMC2680537

[121] Hildebrandt R, Weitzel H, Warnke K, Gundert-Remy U. Pharmacokinetics of fenoterol in pregnant and nonpregnant women. Eur J Clin Pharmacol. 1993;45(3):275–7. 8276054 10.1007/BF00315396

[122] Peiker G, Traeger A, Stiller K, Muller B. [Pharmacokinetics and electrolyte balance following administration of furosemide in pregnant women with E gestosis]. Pharmazie. 1984;39(5):336–9. 6473496

[123] Fischer JH, Sarto GE, Hardman J, Endres L, Jenkins TM, Kilpatrick SJ, et al Influence of gestational age and body weight on the pharmacokinetics of labetalol in pregnancy. Clin Pharmacokinet. 2014;53(4):373–83. 10.1007/s40262-013-0123-0 24297680 PMC4310214

[124] Rubin PC, Butters L, Kelman AW, Fitzsimons C, Reid JL. Labetalol disposition and concentration-effect relationships during pregnancy. Br J Clin Pharmacol. 1983;15(4):465–70. 6849783 10.1111/j.1365-2125.1983.tb01531.x PMC1427790

[125] Rogers RC, Sibai BM, Whybrew WD. Labetalol pharmacokinetics in pregnancy-induced hypertension. Am J Obstet Gynecol. 1990;162(2):362–6. 2309815 10.1016/0002-9378(90)90386-l

[126] Gonser M, Stoll P, Kahle P. Clearance prediction and drug dosage in pregnancy: a clinical study on metildigoxin, and application to other drugs with predominant penal elimination. Clin Drug Investig. 1995;9(4):197–205.

[127] Hogstedt S, Rane A. Plasma concentration-effect relationship of metoprolol during and after pregnancy. Eur J Clin Pharmacol. 1993;44(3):243–6. 8491238 10.1007/BF00271365

[128] Prevost RR, Akl SA, Whybrew WD, Sibai BM. Oral nifedipine pharmacokinetics in pregnancy-induced hypertension. Pharmacotherapy. 1992;12(3):174–7. 1608848

[129] O’Hare MF, Leahey W, Murnaghan GA, McDevitt DG. Pharmacokinetics of sotalol during pregnancy. Eur J Clin Pharmacol. 1983;24(4):521–4. 6861867 10.1007/BF00609896

[130] Best BM, Mirochnick M, Capparelli EV, Stek A, Burchett SK, Holland DT, et al Impact of pregnancy on abacavir pharmacokinetics. AIDS. 2006;20(4):553–60. 10.1097/01.aids.0000210609.52836.d1 16470119

[131] Conradie F, Zorrilla C, Josipovic D, Botes M, Osiyemi O, Vandeloise E, et al Safety and exposure of once-daily ritonavir-boosted atazanavir in HIV-infected pregnant women. HIV Med. 2011;12(9):570–9. 10.1111/j.1468-1293.2011.00927.x 21569187

[132] Ripamonti D, Cattaneo D, Maggiolo F, Airoldi M, Frigerio L, Bertuletti P, et al Atazanavir plus low-dose ritonavir in pregnancy: pharmacokinetics and placental transfer. AIDS. 2007;21(18):2409–15. 10.1097/QAD.0b013e32825a69d1 18025877

[133] Kreitchmann R, Best BM, Wang J, Stek A, Caparelli E, Watts DH, et al Pharmacokinetics of an increased atazanavir dose with and without tenofovir during the third trimester of pregnancy. J Acquire Immune Defic Syndr. 2013;63(1):59–66. 10.1097/QAI.0b013e318289b4d2 PMC3625451 23392467

[134] Mirochnick M, Best BM, Stek AM, Capparelli EV, Hu C, Burchett SK, et al Atazanavir pharmacokinetics with and without tenofovir during pregnancy. J Acquire Immune Defic Syndr. 2011;56(5):412–9. 10.1097/QAI.0b013e31820fd093 PMC3125419 21283017

[135] Else LJ, Jackson V, Brennan M, Back DJ, Khoo SH, Coulter-Smith S, et al Therapeutic drug monitoring of atazanavir/ritonavir in pregnancy. HIV Med. 2014;15(10):604–10. 10.1111/hiv.12164 24825070

[136] Colbers A, Hawkins D, Hidalgo-Tenorio C, van der Ende M, Gingelmaier A, Weizsacker K, et al Atazanavir exposure is effective during pregnancy regardless of tenofovir use. Antivir Ther. 2015;20(1):57–64. 10.3851/IMP2820 24992294

[137] Le MP, Mandelbrot L, Descamps D, Soulie C, Ichou H, Bourgeois-Moine A, et al Pharmacokinetics, safety and efficacy of ritonavir-boosted atazanavir (300/100 mg once daily) in HIV-1-infected pregnant women. Antivir Ther. 2015;20(5):507–13. 10.3851/IMP2936 25599649

[138] Colbers A, Molto J, Ivanovic J, Kabeya K, Hawkins D, Gingelmaier A, et al Pharmacokinetics of total and unbound darunavir in HIV-1-infected pregnant women. J Antimicrob Chemother. 2015;70(2):534–42. 10.1093/jac/dku400 25326090

[139] Zorrilla CD, Wright R, Osiyemi O, Yasin S, Baugh B, Brown K, et al Total and unbound darunavir pharmacokinetics in pregnant women infected with HIV-1: results of a study of darunavir/ritonavir 600/100mg administered twice daily. HIV Med. 2014;15(1):50–6. 10.1111/hiv.12047 23731450 PMC4231999

[140] Stek A, Best BM, Wang J, Capparelli EV, Burchett SK, Kreitchmann R, et al Pharmacokinetics of once versus twice daily darunavir in pregnant HIV-infected women. J Acquire Immune Defic Syndr. 2015;70(1):33–41. 10.1097/QAI.0000000000000668 PMC4579345 25950206

[141] Wang Y, Livingston E, Patil S, McKinney RE, Bardeguez AD, Gandia J, et al Pharmacokinetics of didanosine in antepartum and postpartum human immunodeficiency virus—infected pregnant women and their neonates: an AIDS clinical trials group study. J Infect Dis. 1999;180(5):1536–41. 10.1086/315067 10515813

[142] Bartelink IH, Savic RM, Mwesigwa J, Achan J, Clark T, Plenty A, et al Pharmacokinetics of lopinavir/ritonavir and efavirenz in food insecure HIV-infected pregnant and breastfeeding women in Tororo, Uganda. J Clin Pharmacol. 2014;54(2):121–32. 10.1002/jcph.167 24038035 PMC3933454

[143] Cressey TR, Stek A, Capparelli E, Bowonwatanuwong C, Prommas S, Sirivatanapa P, et al Efavirenz pharmacokinetics during the third trimester of pregnancy and postpartum. J Acquire Immune Defic Syndr. 2012;59(3):245–52. 10.1097/QAI.0b013e31823ff052 PMC3288559 22083071

[144] Olagunju A, Bolaji O, Amara A, Else L, Okafor O, Adejuyigbe E, et al Pharmacogenetics of pregnancy-induced changes in efavirenz pharmacokinetics. Clin Pharmacol Ther. 2015;97(3):298–306. 10.1002/cpt.43 25669165

[145] Colbers APH, Hawkins DA, Gingelmaier A, Kabeya K, Rockstroh JK, Wyen C, et al The pharmacokinetics, safety and efficacy of tenofovir and emtricitabine in HIV-1-infected pregnant women. AIDS. 2013;27(5):739–48. 10.1097/QAD.0b013e32835c208b 23169329

[146] Stek AM, Best BM, Luo W, Capparelli E, Burchett S, Hu C, et al Effect of pregnancy on emtricitabine pharmacokinetics. HIV Med. 2012;13(4):226–35. 10.1111/j.1468-1293.2011.00965.x 22129166 PMC3342997

[147] Valade E, Treluyer JM, Dabis F, Arrive E, Pannier E, Benaboud S, et al Modified renal function in pregnancy: impact on emtricitabine pharmacokinetics. Br J Clin Pharmacol. 2014;78(6):1378–86. 10.1111/bcp.12457 24995851 PMC4256626

[148] Cressey TR, Best BM, Achalapong J, Stek A, Wang J, Chotivanich N, et al Reduced indinavir exposure during pregnancy. Br J Clin Pharmacol. 2013;76(3):475–83. 10.1111/bcp.12078 23305215 PMC3769674

[149] Kosel BW, Beckerman KP, Hayashi S, Homma M, Aweeka FT. Pharmacokinetics of nelfinavir and indinavir in HIV-1-infected pregnant women. AIDS. 2003;17(8):1195–9. 10.1097/01.aids.0000060367.78202.cb 12819521

[150] Unadkat JD, Wara DW, Hughes MD, Mathias AA, Holland DT, Paul ME, et al Pharmacokinetics and safety of indinavir in human immunodeficiency virus-infected pregnant women. Antimicrob Agents Chemother. 2007;51(2):783–6. 10.1128/AAC.00420-06 17158945 PMC1797783

[151] Benaboud S, Treluyer JM, Urien S, Blanche S, Bouazza N, Chappuy H, et al Pregnancy-related effects on lamivudine pharmacokinetics in a population study with 228 women. Antimicrob Agents Chemother. 2012;56(2):776–82. 10.1128/AAC.00370-11 22106227 PMC3264256

[152] Bouillon-Pichault M, Jullien V, Azria E, Pannier E, Firtion G, Krivine A, et al Population analysis of the pregnancy-related modifications in lopinavir pharmacokinetics and their possible consequences for dose adjustment. J Antimicrob Chemother. 2009;63(6):1223–32. 10.1093/jac/dkp123 19389715

[153] Calza L, Manfredi R, Trapani F, Salvadori C, Colangeli V, Borderi M, et al Lopinavir/ritonavir trough concentrations with the tablet formulation in HIV-1-infected women during the third trimester of pregnancy. Scand J Infect Dis. 2012;44(5):381–7. 10.3109/00365548.2011.642306 22263609

[154] Else LJ, Douglas M, Dickinson L, Back DJ, Khoo SH, Taylor GP. Improved oral bioavailability of lopinavir in melt-extruded tablet formulation reduces impact of third trimester on lopinavir plasma concentrations. Antimicrob Agents Chemother. 2012;56(2):816–24. 10.1128/AAC.05186-11 22106215 PMC3264219

[155] Lambert JS, Else LJ, Jackson V, Breiden J, Gibbons S, Dickinson L, et al Therapeutic drug monitoring of lopinavir/ritonavir in pregnancy. HIV Med. 2011;12(3):166–73. 10.1111/j.1468-1293.2010.00865.x 20726906

[156] Mirochnick M, Best BM, Stek AM, Capparelli E, Hu C, Burchett SK, et al Lopinavir exposure with an increased dose during pregnancy. J Acquire Immune Defic Syndr. 2008;49(5):485–91. 10.1097/QAI.0b013e318186edd0 PMC3912695 18989231

[157] Ramautarsing RA, Van Der Lugt J, Gorowara M, Kerr SJ, Burger D, Ruxrungtham K, et al Thai HIV-1-infected women do not require a dose increase of lopinavir/ritonavir during the third trimester of pregnancy. AIDS. 2011;25(10):1299–303. 10.1097/QAD.0b013e328347f7e9 21516029

[158] Santini-Oliveira M, Estrela Rde C, Veloso VG, Cattani VB, Yanavich C, Velasque L, et al Randomized clinical trial comparing the pharmacokinetics of standard- and increased-dosage lopinavir-ritonavir coformulation tablets in HIV-positive pregnant women. Antimicrob Agents Chemother. 2014;58(5):2884–93. 10.1128/AAC.02599-13 24614377 PMC3993202

[159] Stek AM, Mirochnick M, Capparelli E, Best BM, Hu C, Burchett SK, et al Reduced lopinavir exposure during pregnancy. AIDS. 2006;20(15):1931–9. 10.1097/01.aids.0000247114.43714.90 16988514

[160] Patterson KB, Dumond JB, Prince HA, Jenkins AJ, Scarsi KK, Wang R, et al Protein binding of lopinavir and ritonavir during 4 phases of pregnancy: implications for treatment guidelines. J Acquire Immune Defic Syndr. 2013;63(1):51–8. 10.1097/QAI.0b013e31827fd47e PMC3625477 23221983

[161] Aweeka FT, Stek A, Best BM, Hu C, Holland D, Hermes A, et al Lopinavir protein binding in HIV-1-infected pregnant women. HIV Med. 2010;11(4):232–8. 10.1111/j.1468-1293.2009.00767.x 20002783 PMC2874627

[162] Fauchet F, Treluyer JM, Illamola SM, Pressiat C, Lui G, Valade E, et al Population approach to analyze the pharmacokinetics of free and total lopinavir in HIV-infected pregnant women and consequences for dose adjustment. Antimicrob Agents Chemother. 2015;59(9):5727–35. 10.1128/AAC.00863-15 26149996 PMC4538471

[163] Fang A, Valluri SR, O’Sullivan MJ, Maupin R, Jones T, Delke I, et al Safety and pharmacokinetics of nelfinavir during the second and third trimesters of pregnancy and postpartum. HIV Clin Trials. 2012;13(1):46–59. 10.1310/hct1301-046 22306587

[164] Hirt D, Treluyer JM, Jullien V, Firtion G, Chappuy H, Rey E, et al Pregnancy-related effects on nelfinavir-M8 pharmacokinetics: a population study with 133 women. Antimicrob Agents Chemother. 2006;50(6):2079–86. 10.1128/AAC.01596-05 16723569 PMC1479099

[165] Nellen JFJB, Schillevoort I, Wit FWNM, Bergshoeff AS, Godfried MH, Boer K, et al Nelfinavir plasma concentrations are low during pregnancy. Clin Infect Dis. 2004;39(5):736–40. 10.1086/422719 15356791

[166] Read JS, Best BM, Stek AM, Hu C, Capparelli EV, Holland DT, et al Pharmacokinetics of new 625mg nelfinavir formulation during pregnancy and postpartum. HIV Med. 2008;9(10):875–82. 10.1111/j.1468-1293.2008.00640.x 18795962 PMC2732353

[167] Villani P, Floridia M, Pirillo MF, Cusato M, Tamburrini E, Cavaliere AF, et al Pharmacokinetics of nelfinavir in HIV-1-infected pregnant and nonpregnant women. Br J Clin Pharmacol. 2006;62(3):309–15. 10.1111/j.1365-2125.2006.02669.x 16934047 PMC1885131

[168] Van Heeswijk RPG, Khaliq Y, Gallicano KD, Bourbeau M, Seguin I, Phillips EJ, et al The pharmacokinetics of nelfinavir and M8 during pregnancy and post partum. Clin Pharmacol Ther. 2004;76(6):588–97. 10.1016/j.clpt.2004.08.011 15592330

[169] Capparelli EV, Aweeka F, Hitti J, Stek A, Hu C, Burchett SK, et al Chronic administration of nevirapine during pregnancy: impact of pregnancy on pharmacokinetics. HIV Med. 2008;9(4):214–20. 10.1111/j.1468-1293.2008.00553.x 18366444 PMC2755564

[170] Nellen JFJB, Damming M, Godfried MH, Boer K, Van Der Ende ME, Burger DM, et al Steady-state nevirapine plasma concentrations are influenced by pregnancy. HIV Med. 2008;9(4):234–8. 10.1111/j.1468-1293.2008.00551.x 18366447

[171] Lamorde M, Byakika-Kibwika P, Okaba-Kayom V, Flaherty JP, Boffito M, Namakula R, et al Suboptimal nevirapine steady-state pharmacokinetics during intrapartum compared with postpartum in HIV-1-seropositive Ugandan women. J Acquire Immune Defic Syndr. 2010;55(3):345–50. 10.1097/QAI.0b013e3181e9871b PMC3594885 20622674

[172] Blonk M, Colbers A, Hidalgo-Tenorio C, Weizsacker K, Molto J, Hawkins D, et al A comparison of the pharmacokinetics of raltegravir during pregnancy and postpartum. Top Antivir Med. 2014;22 (e-1):465.

[173] Watts DH, Stek A, Best BM, Wang J, Capparelli EV, Cressey TR, et al Raltegravir pharmacokinetics during pregnancy. J Acquir Immune Defic Syndr. 2014;67(4):375–81. 10.1097/QAI.0000000000000318 25162818 PMC4213295

[174] Martinez-Rebollar M, Lonca M, Perez I, Soy D, Brunet M, Martin R, et al Pharmacokinetic study of saquinavir 500 mg plus ritonavir (1000/100 mg twice a day) in HIV-positive pregnant women. Ther Drug Monit. 2011;33(6):772–7. 10.1097/FTD.0b013e318236376d 22105596

[175] van der Lug J, Colbers A, Molto J, Hawkins D, van der Ende M, Vogel M, et al The pharmacokinetics, safety and efficacy of boosted saquinavir tablets in HIV type-1-infected pregnant women. Antivir Ther. 2009;14(3):443–50. 19474478

[176] von Hentig N, Nisius G, Lennemann T, Khaykin P, Stephan C, Babacan E, et al Pharmacokinetics, safety and efficacy of saquinavir/ritonavir 1,000/100 mg twice daily as HIV type-1 therapy and transmission prophylaxis in pregnancy. Antivir Ther. 2008;13(8):1039–46. 19195329

[177] Cespedes MS, Castor D, Ford SL, Lee D, Lou Y, Pakes GE, et al Steady-state pharmacokinetics, cord blood concentrations, and safety of ritonavir-boosted fosamprenavir in pregnancy. J Acquire Immune Defic Syndr. 2013;62(5):550–4. 10.1097/QAI.0b013e318285d918 PMC3683080 23314414

[178] Green MD, Van Eijk AM, Van Ter Kuile FO, Ayisi JG, Parise ME, Kager PA, et al Pharmacokinetics of sulfadoxine-pyrimethamine in HIV-infected and uninfected pregnant women in western Kenya. J Infect Dis. 2007;196(9):1403–8. 10.1086/522632 17922406

[179] Benaboud S, Hirt D, Launay O, Pannier E, Firtion G, Rey E, et al Pregnancy-related effects on tenofovir pharmacokinetics: A population study with 186 women. Antimicrob Agents Chemother. 2012;56(2):857–62. 10.1128/AAC.05244-11 22123690 PMC3264275

[180] Hirt D, Urien S, Ekouevi DK, Rey E, Arrive E, Blanche S, et al Population pharmacokinetics of tenofovir in HIV-1-infected pregnant women and their neonates (ANRS 12109). Clin Pharmacol Ther. 2009;85(2):182–9. 10.1038/clpt.2008.201 18987623

[181] Mosha D, Guidi M, Mwingira F, Abdulla S, Mercier T, Decosterd LA, et al Population pharmacokinetics and clinical response for artemether-lumefantrine in pregnant and nonpregnant women with uncomplicated Plasmodium falciparum malaria in Tanzania. Antimicrob Agents Chemother. 2014;58(8):4583–92. 10.1128/AAC.02595-14 24867986 PMC4136066

[182] McGready R, Stepniewska K, Lindegardh N, Ashley EA, La Y, Singhasivanon P, et al The pharmacokinetics of artemether and lumefantrine in pregnant women with uncomplicated falciparum malaria. Eur J Clin Pharmacol. 2006;62(12):1021–31. 10.1007/s00228-006-0199-7 17053895

[183] McGready R, Stepniewska K, Edstein MD, Cho T, Gilveray G, Looareesuwan S, et al The pharmacokinetics of atovaquone and proguanil in pregnant women with acute falciparum malaria. Eur J Clin Pharmacol. 2003;59(7):545–52. 10.1007/s00228-003-0652-9 12955371

[184] Chukwuani MC, Bolaji OO, Onyeji CO, Makinde ON, Ogunbona FA. Evidence for increased metabolism of chloroquine during the early third trimester of human pregnancy. Trop Med Int Health. 2004;9(5):601–5. 10.1111/j.1365-3156.2004.01227.x 15117305

[185] Karunajeewa HA, Salman S, Mueller I, Baiwog F, Gomorrai S, Law I, et al Pharmacokinetics of chloroquine and monodesethylchloroquine in pregnancy. Antimicrob Agents Chemother. 2010;54(3):1186–92. 10.1128/AAC.01269-09 20086162 PMC2825967

[186] Lee SJ, McGready R, Fernandez C, Stepniewska K, Paw MK, Viladpai-Nguen SJ, et al Chloroquine pharmacokinetics in pregnant and nonpregnant women with vivax malaria. Eur J Clin Pharmacol. 2008;64(10):987–92. 10.1007/s00228-008-0500-z 18594802

[187] Massele AY, Kilewo C, Aden Abdi Y, Tomson G, Diwan VK, Ericsson O, et al Chloroquine blood concentrations and malaria prophylaxis in Tanzanian women during the second and third trimesters of pregnancy. Eur J Clin Pharmacol. 1997;52(4):299–305. 9248769 10.1007/s002280050294

[188] Kloprogge F, Piola P, Dhorda M, Muwanga S, Turyakira E, Apinan S, et al Population pharmacokinetics of lumefantrine in pregnant and nonpregnant women with uncomplicated plasmodium falciparum malaria in Uganda. CPT Pharmacometrics Syst Pharmacol. 2013;2(11):e83. 24226803 10.1038/psp.2013.59 PMC3852159

[189] Tarning J, Kloprogge F, Dhorda M, Jullien V, Nosten F, White NJ, et al Pharmacokinetic properties of artemether, dihydroartemisinin, lumefantrine, and quinine in pregnant women with uncomplicated plasmodium falciparum malaria in Uganda. Antimicrob Agents Chemother. 2013;57(10):5096–103. 10.1128/AAC.00683-13 23917320 PMC3811434

[190] Nosten F, Karbwang J, White NJ, Honeymoon, Na Bangchang K, Bunnag D, et al Mefloquine antimalarial prophylaxis in pregnancy: dose finding and pharmacokinetic study. Br J Clin Pharmacol. 1990;30(1):79–85. 2390434 10.1111/j.1365-2125.1990.tb03746.x PMC1368278

[191] Bangchang KN, Davis TME, Looareesuwan S, White NJ, Bunnag D, Karbwang J. Mefloquine pharmacokinetics in pregnant women with acute falciparum malaria. Trans R Soc Trop Med Hyg. 1994;88(3):321–3. 7974678 10.1016/0035-9203(94)90101-5

[192] Valea I, Tinto H, Coulibaly MT, Toe LC, Lindegardh N, Tarning J, et al Pharmacokinetics of co-formulated mefloquine and artesunate in pregnant and non-pregnant women with uncomplicated Plasmodium falciparum infection in Burkina Faso. J Antimicrob Chemother. 2014;69(9):2499–507. 10.1093/jac/dku154 24891429 PMC4130382

[193] Rijken MJ, McGready R, Phyo AP, Lindegardh N, Tarning J, Laochan N, et al Pharmacokinetics of dihydroartemisinin and piperaquine in pregnant and nonpregnant women with uncomplicated falciparum malaria. Antimicrob Agents Chemother. 2011;55(12):5500–6. 10.1128/AAC.05067-11 21947392 PMC3232755

[194] Benjamin JM, Moore BR, Salman S, Page-Sharp M, Tawat S, Yadi G, et al Population pharmacokinetics, tolerability, and safety of dihydroartemisinin-piperaquine and sulfadoxine-pyrimethamine-piperaquine in pregnant and nonpregnant Papua New Guinean women. Antimicrob Agents Chemother. 2015;59(7):4260–71. 10.1128/AAC.00326-15 25963981 PMC4468729

[195] Tarning J, Rijken MJ, McGready R, Phyo AP, Hanpithakpong W, Day NPJ, et al Population pharmacokinetics of dihydroartemisinin and piperaquine in pregnant and nonpregnant women with uncomplicated malaria. Antimicrob Agents Chemother. 2012;56(4):1997–2007. 10.1128/AAC.05756-11 22252822 PMC3318332

[196] Wangboonskul J, White NJ, Nosten F, Ter Kuile F, Moody RR, Taylor RB. Single dose pharmacokinetics of proguanil and its metabolites in pregnancy. Eur J Clin Pharmacol. 1993;44(3):247–51. 8491239 10.1007/BF00271366

[197] Onyamboko MA, Meshnick SR, Fleckenstein L, Koch MA, Atibu J, Lokomba V, et al Pharmacokinetics and pharmacodynamics of artesunate and dihydroartemisinin following oral treatment in pregnant women with asymptomatic Plasmodium falciparum infections in Kinshasa DRC. Malar J. 2011;10(49). 10.1186/1475-2875-10-49 PMC3056842 21352601

[198] McGready R, Phyo AP, Rijken MJ, Tarning J, Lindegardh N, Hanpithakpon W, et al Artesunate/dihydroartemisinin pharmacokinetics in acute falciparum malaria in pregnancy: absorption, bioavailability, disposition and disease effects. Br J Clin Pharmacol. 2012;73(3):467–77. 10.1111/j.1365-2125.2011.04103.x 21950338 PMC3370352

[199] Karunajeewa HA, Salman S, Mueller I, Baiwog F, Gomorrai S, Law I, et al Pharmacokinetic properties of sulfadoxine-pyrimethamine in pregnant women. Antimicrob Agents Chemother. 2009;53(10):4368–76. 10.1128/AAC.00335-09 19620325 PMC2764169

[200] Nyunt MM, Adam I, Kayentao K, van Dijk J, Thuma P, Mauff K, et al Pharmacokinetics of sulfadoxine and pyrimethamine in intermittent preventive treatment of malaria in pregnancy. Clin Pharmacol Ther. 2010;87(2):226–34. 10.1038/clpt.2009.177 19776738

[201] Concheiro M, Jones HE, Johnson RE, Choo R, Huestis MA. Preliminary buprenorphine sublingual tablet pharmacokinetic data in plasma, oral fluid, and sweat during treatment of opioid-dependent pregnant women. Ther Drug Monit. 2011;33(5):619–26. 10.1097/FTD.0b013e318228bb2a 21860340 PMC3178674

[202] Bogen DL, Perel JM, Helsel JC, Hanusa BH, Romkes M, Nukui T, et al Pharmacologic evidence to support clinical decision making for peripartum methadone treatment. Psychopharmacology. 2013;225(2):441–51. 10.1007/s00213-012-2833-7 22926004 PMC3537905

[203] Jarvis MAE, Wu-Pong S, Kniseley JS, Schnoll SH. Alterations in methadone metabolism during late pregnancy. J Addict Dis. 1999;18(4):51–61. 10.1300/J069v18n04_05 10631963

[204] Wolff K, Boys A, Rostami-Hodjegan A, Hay A, Raistrick D. Changes to methadone clearance during pregnancy. Eur J Clin Pharmacol. 2005;61(10):763–8. 10.1007/s00228-005-0035-5 16261362

[205] Van Calsteren K, Verbesselt R, Ottevanger N, Halaska M, Heyns L, Van Bree R, et al Pharmacokinetics of chemotherapeutic agents in pregnancy: a preclinical and clinical study. Acta Obstet Gynecol Scand. 2010;89(10):1338–45. 10.3109/00016349.2010.512070 20846067

[206] Zemlickis D, Klein J, Moselhy G, Koren G. Cisplatin protein binding in pregnancy and the neonatal period. Med Pediatr Oncol. 1994;23(6):476–9. 7935173 10.1002/mpo.2950230605

[207] Bjorklund AO, Adamson UKC, Lins PES, Westgren LMR. Diminished insulin clearance during late pregnancy in patients with Type I diabetes mellitus. Clin Sci. 1998;95(3):317–23. 9730851

[208] Charles B, Norris R, Xiao X, Hague W. Population pharmacokinetics of metformin in late pregnancy. Ther Drug Monit. 2006;28(1):67–72. 16418696 10.1097/01.ftd.0000184161.52573.0e

[209] Eyal S, Easterling TR, Carr D, Umans JG, Miodovnik M, Hankins GDV, et al Pharmacokinetics of metformin during pregnancy. Drug Metab Dispos. 2010;38(5):833–40. 10.1124/dmd.109.031245 20118196 PMC2872944

[210] Hughes RCE, Gardiner SJ, Begg EJ, Zhang M. Effect of pregnancy on the pharmacokinetics of metformin. Diabet Med. 2006;23(3):323–6. 10.1111/j.1464-5491.2005.01769.x 16492218

[211] Bajoria R, Oteng-Ntim E, Peek MJ, Fisk NM. Pharmacokinetics and pharmacodynamics of TRH during pregnancy. Obstet Gynecol. 1997;90(2):176–82. 10.1016/S0029-7844(97)00221-4 9241288

[212] Davison JM, Sheills EA, Philips PR, Barron WM, Lindheimer MD. Metabolic clearance of vasopressin and an analogue resistant to vasopressinase in human pregnancy. Am J Physiol Renal Physiol. 1993;264(2 Pt 2):F348–53. 10.1152/ajprenal.1993.264.2.F348 8447444

[213] Caritis SN, Venkataramanan R, Cotroneo M, Chiao JP. Pharmacokinetics of orally administered ritodrine. Am J Obstet Gynecol. 1989;161(1):32–5. 2641984 10.1016/0002-9378(89)90225-1

[214] Berg G, Lindberg C, Ryden G. Terbutaline in the treatment of preterm labour. Eur J Respir Dis Suppl. 1984;134:219–30. 6586483

[215] ter Laak MA, Roos C, Touw DJ, van Hattum PR, Kwee A, Lotgering FK, et al Pharmacokinetics of nifedipine slow-release during sustained tocolysis. Int J Clin Pharmacol Ther. 2015;53(1):84–91. 10.5414/CP202215 25407260

[216] Ryu RJ, Eyal S, Kaplan HG, Akbarzadeh A, Hays K, Puhl K, et al Pharmacokinetics of doxorubicin in pregnant women. Cancer Chemother Pharmacol. 2014;73(4):789–97. 10.1007/s00280-014-2406-z 24531558 PMC3982413

[217] Freeman MP, Nolan PE Jr, Davis MF, Anthony M, Fried K, Fankhauser M, et al Pharmacokinetics of sertraline across pregnancy and postpartum. J Clin Psychopharmacol. 2008;28(6):646–53. 10.1097/JCP.0b013e31818d2048 19011433

[218] O’Hare MF, Kinney CD, Murnaghan GA, McDevitt DG. Pharmacokinetics of propranolol during pregnancy. Eur J Clin Pharmacol. 1984;27(5):583–7. 6519163 10.1007/BF00556896

[219] Smith MT, Livingstone I, Eadie MJ, Hooper WD, Triggs EJ. Chronic propranolol administration during pregnancy. Maternal pharmacokinetics. Eur J Clin Pharmacol. 1983;25(4):481–90. 6653642 10.1007/BF00542115

[220] Abdelrahim II, Adam I, Elghazali G, Gustafsson LL, Elbashir MI, Mirghani RA. Pharmacokinetics of quinine and its metabolites in pregnant Sudanese women with uncomplicated Plasmodium falciparum malaria. J Clin Pharm Ther. 2007;32(1):15–9. 10.1111/j.1365-2710.2007.00788.x 17286785

[221] Shere M, Nguyen P, Tam C, Stern S, Kapur B, O’Connor D, et al Optimizing periconceptional folic acid supplementation: steady-state folate pharmacokinetics in pregnancy. FASEB J. 2014;28:LB416.

[222] Gregory IJF, Caudill MA, Jeffery Opalko F, Bailey LB. Kinetics of folate turnover in pregnant women (second trimester) and nonpregnant controls during folic acid supplementation: stable-isotopic labeling of plasma folate, urinary folate and folate catabolites shows subtle effects of pregnancy on turnover of folate pools. J Nutr. 2001;131(7):1928–37. 11435509 10.1093/jn/131.7.1928

[223] Roth DE, Al Mahmud A, Raqib R, Akhtar E, Black RE, Baqui AH. Pharmacokinetics of high-dose weekly oral vitamin D3 supplementation during the third trimester of pregnancy in Dhaka, Bangladesh. Nutrients. 2013;5(3):788–810. 10.3390/nu5030788 23482056 PMC3705320

[224] Roth DE, Al Mahmud A, Raqib R, Black RE, Baqui AH. Pharmacokinetics of a single oral dose of vitamin D3 (70,000 IU) in pregnant and non-pregnant women. Nutr J. 2012;11:114 10.1186/1475-2891-11-114 23268736 PMC3552819

[225] Voigt R, Schroder S, Bierwisch I, Michels W, Spencker FB. [Pharmacokinetic studies of cefotiam in late pregnancy]. Z Geburtshilfe Perinatol. 1989;193(4):185–7. 2800661

[226] Peiker G, Muller B, Voigt G, Noschel H. [Blood level kinetics of sodium noramidopyrine methansulphonate (Analgin) in late pregnancy]. Pharmazie. 1983;38(6):406–8. 6611643

[227] Guay J, Beaudry B, Lortie L, Varin F. Pharmacokinetics of atracurium in pregnant women. Clin Drug Investig. 1996;11(3):167–73.

[228] Pihlajamaki K, Kanto J, Lindberg R, Karanko M, Kiilholma P. Extradural administration of bupivacaine: pharmacokinetics and metabolism in pregnant and non-pregnant women. Br J Anaesth. 1990;64(5):556–62. 2354094 10.1093/bja/64.5.556

[229] Peiker G, Mueller B, Ihn W, Noeschel H. [The pharmacokinetics of pethidin (Dolcontral) of parturients and their new-borns]. Pharmazie. 1981;36(8):560–3. 7291290

[230] Elkomy MH, Sultan P, Carvalho B, Peltz G, Wu M, Clavijo C, et al Ondansetron pharmacokinetics in pregnant women and neonates: towards a new treatment for neonatal abstinence syndrome. Clin Pharmacol Ther. 2015;97(2):167–76. 10.1002/cpt.5 25670522 PMC4325425

[231] O’Sullivan MJ, Boyer PJJ, Scott GB, Parks WP, Weller S, Blum R, et al The pharmacokinetics and safety of zidovudine in the third trimester of pregnancy for women infected with human immunodeficiency virus and their infants: phase I Acquired Immunodeficiency Syndrome Clinical Trials Group study (protocol 082). Am J Obstet Gynecol. 1993;168(5):1510–6. 8098905 10.1016/s0002-9378(11)90791-1

[232] Shi YE, Ye ZH, He CH, Zhang GQ, Xu JQ, Van Look PFA, et al Pharmacokinetic study of RU 486 and its metabolites after oral administration of single doses to pregnant and non-pregnant women. Contraception. 1993;48(2):133–49. 8403910 10.1016/0010-7824(93)90004-q

[233] Gardner DF, Cruikshank DP, Hays PM, Cooper DS. Pharmacology of propylthiouracil (PTU) in pregnant hyperthyroid women: correlation of maternal PTU concentrations with cord serum thyroid function tests. J Clin Endocrinol Metab. 1986;62(1):217–20. 10.1210/jcem-62-1-217 3940267

[234] Chopra IJ, Baber K. Treatment of primary hypothyroidism during pregnancy: is there an increase in thyroxine dose requirement in pregnancy? Metabolism. 2003;52(1):122–8. 10.1053/meta.2003.50019 12524672

[235] Ensom MHH, Stephenson MD. A two-center study on the pharmacokinetics of intravenous immunoglobulin before and during pregnancy in healthy women with poor obstetrical histories. Hum Reprod. 2011;26(9):2283–8. 10.1093/humrep/der227 21771770 PMC3157629

[236] Rijken MJ, McGready R, Jullien V, Tarning J, Lindegardh N, Phyo AP, et al Pharmacokinetics of amodiaquine and desethylamodiaquine in pregnant and postpartum women with Plasmodium vivax malaria. Antimicrob Agents Chemother. 2011;55(9):4338–42. 10.1128/AAC.00154-11 21709098 PMC3165320

[237] Kloprogge F, Jullien V, Piola P, Dhorda M, Muwanga S, Nosten F, et al Population pharmacokinetics of quinine in pregnant women with uncomplicated Plasmodium falciparum malaria in Uganda. J Antimicrob Chemother. 2014;69(11):3033–40. 10.1093/jac/dku228 24970740 PMC4195470

[238] Goodwin TM, Millar L, North L, Abrams LS, Weglein RC, Holland ML. The pharmacokinetics of the oxytocin antagonist atosiban in pregnant-women with preterm uterine contractions. Am J Obstet Gynecol. 1995;173(3):913–7. 7573268 10.1016/0002-9378(95)90365-8

[239] Leake RD, Weitzman RE, Fisher DA. Pharmacokinetics of oxytocin in the human subject. Obstet Gynecol. 1980;56(6):701–4. 7443113

[240] Hutchings MJ, Paull JD, Wilson-Evered E, Morgan DJ. Pharmacokinetics and metabolism of salbutamol in premature labour. Br J Clin Pharmacol. 1987;24(1):69–75. 3620288 10.1111/j.1365-2125.1987.tb03138.x PMC1386282

[241] Peiker G, Muller B, Dawczynski H, Erdmann M, Scharke U. [Comparison of the pharmacokinetics of iron in pregnant and nonpregnant women after oral administration of Vitaferro]. Pharmazie. 1988;43(5):335–6. 3174811

[242] Frenkel LM, Brown ZA, Bryson YJ, Corey L, Unadkat JD, Hensleigh PA, et al Pharmacokinetics of acyclovir in the term human pregnancy and neonate. Am J Obstet Gynecol. 1991;164(2):569–76. 1847004 10.1016/s0002-9378(11)80023-2

[243] Ventura SJ, Curtin SC, Abma JC, Henshaw SK. Estimated pregnancy rates and rates of pregnancy outcomes for the United States, 1990–2008. Natl Vital Stat Rep. 2012;60(7):1–21. 22970648

[244] Yogev Y, Melamed N, Bardin R, Tenenbaum-Gavish K, Ben-Shitrit G, Ben-Haroush A. Pregnancy outcome at extremely advanced maternal age. Am J Obstet Gynecol. 2010;203(6):558.e1–7. 20965486 10.1016/j.ajog.2010.07.039

[245] Thorpe PG, Gilboa SM, Hernandez-Diaz S, Lind J, Cragan JD, Briggs G, et al Medications in the first trimester of pregnancy: most common exposures and critical gaps in understanding fetal risk. Pharmacoepidemiol Drug Saf. 2013;22(9):1013–8. 10.1002/pds.3495 23893932 PMC3996804

[246] McDade TW, Williams S, Snodgrass JJ. What a drop can do: dried blood spots as a minimally invasive method for integrating biomarkers into population-based research. Demography. 2007;44(4):899–925. 18232218 10.1353/dem.2007.0038

[247] Morris CA, Duparc S, Borghini-Fuhrer I, Jung D, Shin CS, Fleckenstein L. Review of the clinical pharmacokinetics of artesunate and its active metabolite dihydroartemisinin following intravenous, intramuscular, oral or rectal administration. Malar J. 2011;10:263 10.1186/1475-2875-10-263 21914160 PMC3180444

[248] Chan WS, Rey E, Kent NE, VTE in Pregnancy Guideline Working Group. Venous thromboembolism and antithrombotic therapy in pregnancy. J Obstet Gynaecol Can. 2014;36(6):527–53. 24927193 10.1016/s1701-2163(15)30569-7

