# Supplementary material for: Pregnancy-Associated Changes in Pharmacokinetics: A Systematic Review
Source: PLoS Med. 2016 Nov 1;13(11):e1002160. doi: 10.1371/journal.pmed.1002160 (PMC5089741; doi:10.1371/journal.pmed.1002160)
Supplement: S1 Table — (DOCX) [file pmed.1002160.s002.docx]

**MEDLINE(R) 1946 to Present [Ovid]**

| 1 | pregnancy trimesters/ or pregnancy trimester, first/ or pregnancy trimester, second/ or pregnancy trimester, third/ |
| --- | --- |
| 2 | Pregnancy/ |
| 3 | ((pregnancies or pregnancy) adj2 (trimester* or midtrimester* or late)).tw. |
| 4 | (trimester* adj2 (first or second or "2nd" or last or third)).tw. |
| 5 | or/1-4 |
| 6 | exp animals/ not humans.sh. |
| 7 | 5 not 6 |
| 8 | pharmacokinetics/ or area under curve/ or biological availability/ or metabolic clearance rate/ or therapeutic equivalency/ or tissue distribution/ |
| 9 | (pharmacokinetic* or "area under curve" or "area under curves" or "area under the curve" or "auc" or "availabilities biologic" or "availabilities physiologic" or "availability biologic" or "availability biological" or "availability equivalencies" or "availability equivalency" or "availability physiologic" or "bioavailabilities" or "bioavailability" or "bioequivalence" or "bioequivalences" or "bioequivalency" or "biologic availabilities" or "biologic availability" or "biological availabilities" or "biological availability" or "biological equivalence" or "biological equivalency" or "clearance rate metabolic" or "clearance rates metabolic" or "clinical equivalencies" or "clinical equivalency" or "cmax" or "distribution kinetics" or "distribution, drug" or "drug absorption" or "drug accumulation" or "drug activation" or "drug adsorption" or "drug availability" or "drug bioavailability" or "drug clearance" or "drug concentration versus time curve" or "drug concentration vs time curve" or "drug concentration- time curve" or "drug degradation" or "drug dialysability" or "drug diffusion" or "drug disposition" or "drug distribution" or "drug elimination" or "drug excretion" or "drug half life" or "drug halflife" or "drug hydrolysis" or "drug inactivation" or "drug incorporation" or "drug localization" or "drug loss" or "drug metabolism " or "drug oxidation" or "drug penetration" or "drug permeability" or "drug plasma half life" or "drug release" or "drug resorption" or "drug retention" or "drug secretion" or "drug sequestration" or "drug transfer" or "drug transformation" or "equivalencies availability" or "equivalencies clinical" or "equivalencies generic" or "equivalencies therapeutic" or "equivalency availability" or "equivalency clinical" or "equivalency generic" or "equivalency therapeutic" or "first pass effect" or "generic equivalencies" or "generic equivalency" or "plasma concentration*" or "serum concentration*" or "mean residence time" or "metabolic clearance rate" or "metabolic clearance rates" or "physiologic availabilities" or "physiologic availability" or "plasma concentration*" or "serum concentration*" or "tablet disintegration" or "therapeutic equivalencies" or "therapeutic equivalency" or "tmax" or "total body clearance rate").tw. |
| 10 | pk.fs. |
| 11 | or/8-10 |
| 12 | 7 and 11 |
| 13 | clinical trial/ or clinical trial, phase i/ or clinical trial, phase ii/ or clinical trial, phase iii/ or clinical trial, phase iv/ or controlled clinical trial/ or multicenter study/ or observational study/ or randomized controlled trial/ or pragmatic clinical trial/ or comparative study/ |
| 14 | case series.mp. |
| 15 | case-control studies/ or retrospective studies/ or cohort studies/ or longitudinal studies/ or follow-up studies/ or prospective studies/ or cross-sectional studies/ or intervention studies/ or pilot projects/ or sampling studies/ |
| 16 | ((multicenter or multicentre or "multi center" or "multi centre" or "phase 1" or "phase i" or "phase 2" or "phase ii" or "phase 3" or "phase iii" or "phase 4" or "phase iv" or "case control*" or intervent* or longitudinal or prospective or retrospective or case or pilot or observational or cohort or sampling or cross section* or follow-up) adj2 (trial or trials or study or studies)).tw. |
| 17 | ("controlled clinical comparison" or "controlled clinical experiment" or "controlled clinical study" or "controlled clinical test" or "control* trial" or "control* group" or "control* study").tw. |
| 18 | ("controlled clinical comparison" or "controlled clinical experiment" or "controlled clinical study" or "controlled clinical test").tw. |
| 19 | ("matched case control*" or "longitudinal evaluation").tw. |
| 20 | ("longitudinal survey" or "prospective method" or "ex post facto design" or "retrospective design" or "case series" or "pilot projects" or "cohort analyses").tw. |
| 21 | or/13-20 |
| **22** | **12 and 21** |

**Embase Classic+Embase 1947 – [Ovid]**

| 1 | pregnancy/ or first trimester pregnancy/ or second trimester pregnancy/ or third trimester pregnancy/ |
| --- | --- |
| 2 | ((pregnancies or pregnancy) adj2 (trimester* or midtrimester* or late)).tw. |
| 3 | (trimester* adj2 (first or second or "2nd" or last or third)).tw. |
| 4 | or/1-3 |
| 5 | exp Animals/ not humans/ |
| 6 | 4 not 5 |
| 7 | exp pharmacokinetics/ |
| 8 | (pharmacokinetic* or "area under curve" or "area under curves" or "area under the curve" or "auc" or "availabilities biologic" or "availabilities physiologic" or "availability biologic" or "availability biological" or "availability equivalencies" or "availability equivalency" or "availability physiologic" or "bioavailabilities" or "bioavailability" or "bioequivalence" or "bioequivalences" or "bioequivalency" or "biologic availabilities" or "biologic availability" or "biological availabilities" or "biological availability" or "biological equivalence" or "biological equivalency" or "clearance rate metabolic" or "clearance rates metabolic" or "clinical equivalencies" or "clinical equivalency" or "cmax" or "distribution kinetics" or "distribution, drug" or "drug absorption" or "drug accumulation" or "drug activation" or "drug adsorption" or "drug availability" or "drug bioavailability" or "drug clearance" or "drug concentration versus time curve" or "drug concentration vs time curve" or "drug concentration- time curve" or "drug degradation" or "drug dialysability" or "drug diffusion" or "drug disposition" or "drug distribution" or "drug elimination" or "drug excretion" or "drug half life" or "drug halflife" or "drug hydrolysis" or "drug inactivation" or "drug incorporation" or "drug localization" or "drug loss" or "drug metabolism " or "drug oxidation" or "drug penetration" or "drug permeability" or "drug plasma half life" or "drug release" or "drug resorption" or "drug retention" or "drug secretion" or "drug sequestration" or "drug transfer" or "drug transformation" or "equivalencies availability" or "equivalencies clinical" or "equivalencies generic" or "equivalencies therapeutic" or "equivalency availability" or "equivalency clinical" or "equivalency generic" or "equivalency therapeutic" or "first pass effect" or "generic equivalencies" or "generic equivalency" or "plasma concentration*" or "serum concentration*" or "mean residence time" or "metabolic clearance rate" or "metabolic clearance rates" or "physiologic availabilities" or "physiologic availability" or "plasma concentration*" or "serum concentration*" or "tablet disintegration" or "therapeutic equivalencies" or "therapeutic equivalency" or "tmax" or "total body clearance rate").tw. |
| 9 | pk.fs. |
| 10 | or/7-9 |
| 11 | 6 and 10 |
| 12 | clinical trial/ or multicenter study/ or phase 1 clinical trial/ or phase 2 clinical trial/ or phase 3 clinical trial/ or phase 4 clinical trial/ |
| 13 | controlled study/ or case control study/ or controlled clinical trial/ or randomized controlled trial/ |
| 14 | exp comparative study/ or observational study/ or pilot study/ |
| 15 | case control study/ or intervention study/ or longitudinal study/ or prospective study/ or retrospective study/ |
| 16 | cross-sectional study/ |
| 17 | follow up/ |
| 18 | cohort analysis/ |
| 19 | ((multicenter or multicentre or "multi center" or "multi centre" or "phase 1" or "phase i" or "phase 2" or "phase ii" or "phase 3" or "phase iii" or "phase 4" or "phase iv" or "case control*" or intervent* or longitudinal or prospective or retrospective or case or pilot or observational or cohort or sampling or cross section* or follow-up) adj2 (trial or trials or study or studies)).tw. |
| 20 | ("controlled clinical comparison" or "controlled clinical experiment" or "controlled clinical study" or "controlled clinical test" or "control* trial" or "control* group" or "control* study").tw. |
| 21 | ("controlled clinical comparison" or "controlled clinical experiment" or "controlled clinical study" or "controlled clinical test").tw. |
| 22 | ("matched case control*" or "longitudinal evaluation").tw. |
| 23 | ("longitudinal survey" or "prospective method" or "ex post facto design" or "retrospective design" or "case series" or "pilot projects" or "cohort analyses").tw. |
| 24 | or/12-23 |
| **25** | **11 and 24** |

**Cochrane Central Register of Controlled Trials [Ovid]**

| 1 | pregnancy trimesters/ or pregnancy trimester, first/ or pregnancy trimester, second/ or pregnancy trimester, third/ |
| --- | --- |
| 2 | Pregnancy/ |
| 3 | ((pregnancies or pregnancy) adj2 (trimester* or midtrimester* or late)).tw. |
| 4 | (trimester* adj2 (first or second or "2nd" or last or third)).tw. |
| 5 | or/1-4 |
| 6 | exp animals/ not humans.sh. |
| 7 | 5 not 6 |
| 8 | pharmacokinetics/ or area under curve/ or biological availability/ or metabolic clearance rate/ or therapeutic equivalency/ or tissue distribution/ |
| 9 | (pharmacokinetic* or "area under curve" or "area under curves" or "area under the curve" or "auc" or "availabilities biologic" or "availabilities physiologic" or "availability biologic" or "availability biological" or "availability equivalencies" or "availability equivalency" or "availability physiologic" or "bioavailabilities" or "bioavailability" or "bioequivalence" or "bioequivalences" or "bioequivalency" or "biologic availabilities" or "biologic availability" or "biological availabilities" or "biological availability" or "biological equivalence" or "biological equivalency" or "clearance rate metabolic" or "clearance rates metabolic" or "clinical equivalencies" or "clinical equivalency" or "cmax" or "distribution kinetics" or "distribution, drug" or "drug absorption" or "drug accumulation" or "drug activation" or "drug adsorption" or "drug availability" or "drug bioavailability" or "drug clearance" or "drug concentration versus time curve" or "drug concentration vs time curve" or "drug concentration- time curve" or "drug degradation" or "drug dialysability" or "drug diffusion" or "drug disposition" or "drug distribution" or "drug elimination" or "drug excretion" or "drug half life" or "drug halflife" or "drug hydrolysis" or "drug inactivation" or "drug incorporation" or "drug localization" or "drug loss" or "drug metabolism " or "drug oxidation" or "drug penetration" or "drug permeability" or "drug plasma half life" or "drug release" or "drug resorption" or "drug retention" or "drug secretion" or "drug sequestration" or "drug transfer" or "drug transformation" or "equivalencies availability" or "equivalencies clinical" or "equivalencies generic" or "equivalencies therapeutic" or "equivalency availability" or "equivalency clinical" or "equivalency generic" or "equivalency therapeutic" or "first pass effect" or "generic equivalencies" or "generic equivalency" or "plasma concentration*" or "serum concentration*" or "mean residence time" or "metabolic clearance rate" or "metabolic clearance rates" or "physiologic availabilities" or "physiologic availability" or "plasma concentration*" or "serum concentration*" or "tablet disintegration" or "therapeutic equivalencies" or "therapeutic equivalency" or "tmax" or "total body clearance rate").tw. |
| 10 | pk.fs. |
| 11 | or/8-10 |
| 12 | 7 and 11 |
| 13 | clinical trial/ or clinical trial, phase i/ or clinical trial, phase ii/ or clinical trial, phase iii/ or clinical trial, phase iv/ or controlled clinical trial/ or multicenter study/ or observational study/ or randomized controlled trial/ or pragmatic clinical trial/ or comparative study/ |
| 14 | case series.mp. |
| 15 | case-control studies/ or retrospective studies/ or cohort studies/ or longitudinal studies/ or follow-up studies/ or prospective studies/ or cross-sectional studies/ or intervention studies/ or pilot projects/ or sampling studies/ |
| 16 | ((multicenter or multicentre or "multi center" or "multi centre" or "phase 1" or "phase i" or "phase 2" or "phase ii" or "phase 3" or "phase iii" or "phase 4" or "phase iv" or "case control*" or intervent* or longitudinal or prospective or retrospective or case or pilot or observational or cohort or sampling or cross section* or follow-up) adj2 (trial or trials or study or studies)).tw. |
| 17 | ("controlled clinical comparison" or "controlled clinical experiment" or "controlled clinical study" or "controlled clinical test" or "control* trial" or "control* group" or "control* study").tw. |
| 18 | ("controlled clinical comparison" or "controlled clinical experiment" or "controlled clinical study" or "controlled clinical test").tw. |
| 19 | ("matched case control*" or "longitudinal evaluation").tw. |
| 20 | ("longitudinal survey" or "prospective method" or "ex post facto design" or "retrospective design" or "case series" or "pilot projects" or "cohort analyses").tw. |
| 21 | or/13-20 |
| **22** | **12 and 21** |

**Web of Science [Thomson Reuters]**

Science Citation Index Expanded (SCI-EXPANDED) --1900-present

Social Sciences Citation Index (SSCI) --1956-present

Conference Proceedings Citation Index- Science (CPCI-S) --1990-present

Conference Proceedings Citation Index- Social Science & Humanities (CPCI-SSH) --1990-present

| **Set** |  |
| --- | --- |
| # 13 | #12 AND #6  *Indexes=SCI-EXPANDED, SSCI, CPCI-S, CPCI-SSH Timespan=All years* |
| # 12 | #11 OR #10 OR #9 OR #8 OR #7  *Indexes=SCI-EXPANDED, SSCI, CPCI-S, CPCI-SSH Timespan=All years* |
| # 11 | TS=("longitudinal survey" or "prospective method" or "ex post facto design" or "retrospective design" or "case series" or "pilot projects" or "cohort analyses" or "cohort analysis")  *Indexes=SCI-EXPANDED, SSCI, CPCI-S, CPCI-SSH Timespan=All years* |
| # 10 | TS=("matched case control*" or "longitudinal evaluation")  *Indexes=SCI-EXPANDED, SSCI, CPCI-S, CPCI-SSH Timespan=All years* |
| # 9 | TS=("controlled clinical comparison" or "controlled clinical experiment" or "controlled clinical study" or "controlled clinical test" or "control* trial" or "control* group" or "control* study")  *Indexes=SCI-EXPANDED, SSCI, CPCI-S, CPCI-SSH Timespan=All years* |
| # 8 | TS=("clinical trial*" or "clinical drug trial*" or "controlled trial*")  *Indexes=SCI-EXPANDED, SSCI, CPCI-S, CPCI-SSH Timespan=All years* |
| # 7 | TS=((multicenter or multicentre or "multi center" or "multi centre" or "phase 1" or "phase i" or "phase 2" or "phase ii" or "phase 3" or "phase iii" or "phase 4" or "phase iv" or "case control*" or intervent* or longitudinal or prospective or retrospective or case or pilot or observational or cohort or sampling or "cross section*" or "follow-up") NEAR/2 (trial or trials or study or studies))  *Indexes=SCI-EXPANDED, SSCI, CPCI-S, CPCI-SSH Timespan=All years* |
| # 6 | #5 AND #4  *Indexes=SCI-EXPANDED, SSCI, CPCI-S, CPCI-SSH Timespan=All years* |
| # 5 | TS=(pharmacokinetic* or "area under curve" or "area under curves" or "area under the curve" or "auc" or "availabilities biologic" or "availabilities physiologic" or "availability biologic" or "availability biological" or "availability equivalencies" or "availability equivalency" or "availability physiologic" or "bioavailabilities" or "bioavailability" or "bioequivalence" or "bioequivalences" or "bioequivalency" or "biologic availabilities" or "biologic availability" or "biological availabilities" or "biological availability" or "biological equivalence" or "biological equivalency" or "clearance rate metabolic" or "clearance rates metabolic" or "clinical equivalencies" or "clinical equivalency" or "cmax" or "distribution kinetics" or "distribution, drug" or "drug absorption" or "drug accumulation" or "drug activation" or "drug adsorption" or "drug availability" or "drug bioavailability" or "drug clearance" or "drug concentration versus time curve" or "drug concentration vs time curve" or "drug concentration- time curve" or "drug degradation" or "drug dialysability" or "drug diffusion" or "drug disposition" or "drug distribution" or "drug elimination" or "drug excretion" or "drug half life" or "drug halflife" or "drug hydrolysis" or "drug inactivation" or "drug incorporation" or "drug localization" or "drug localisation" or "drug loss" or "drug metabolism " or "drug oxidation" or "drug penetration" or "drug permeability" or "drug plasma half life" or "drug release" or "drug resorption" or "drug retention" or "drug secretion" or "drug sequestration" or "drug transfer" or "drug transformation" or "equivalencies availability" or "equivalencies clinical" or "equivalencies generic" or "equivalencies therapeutic" or "equivalency availability" or "equivalency clinical" or "equivalency generic" or "equivalency therapeutic" or "first pass effect" or "generic equivalencies" or "generic equivalency" or "plasma concentration*" or "serum concentration*" or "mean residence time" or "metabolic clearance rate" or "metabolic clearance rates" or "physiologic availabilities" or "physiologic availability" or "plasma concentration*" or "serum concentration*" or "tablet disintegration" or "therapeutic equivalencies" or "therapeutic equivalency" or "tmax" or "total body clearance rate")  *Indexes=SCI-EXPANDED, SSCI, CPCI-S, CPCI-SSH Timespan=All years* |
| # 4 | #3 OR #2 OR #1  *Indexes=SCI-EXPANDED, SSCI, CPCI-S, CPCI-SSH Timespan=All years* |
| # 3 | TS=(trimester* NEAR/2 (first or second or "2nd" or last or third))  *Indexes=SCI-EXPANDED, SSCI, CPCI-S, CPCI-SSH Timespan=All years* |
| # 2 | TS=((pregnancies or pregnancy) NEAR/2 (trimester* or midtrimester* or late))  *Indexes=SCI-EXPANDED, SSCI, CPCI-S, CPCI-SSH Timespan=All years* |
| # 1 | TS=pregnant  *Indexes=SCI-EXPANDED, SSCI, CPCI-S, CPCI-SSH Timespan=All years* |
