# Supplementary material for: Pregnancy-Associated Changes in Pharmacokinetics: A Systematic Review
Source: PLoS Med. 2016 Nov 1;13(11):e1002160. doi: 10.1371/journal.pmed.1002160 (PMC5089741; doi:10.1371/journal.pmed.1002160)
Supplement: S3 Table — (DOCX) [file pmed.1002160.s004.docx]

|  |  |  | **Inclusion and exclusion criteria (0=no, 1=yes)** | | | | | |  |
| --- | --- | --- | --- | --- | --- | --- | --- | --- | --- |
| **Manus ID** | **Citation** | **First Author** | **Human study?** | **PK parameter in pregnancy reported?** | **RCT, cohort, case control, case series?** | **Comparison group (non-pregnant)** | **Include?** | **Comments** | **Drug** |
| **4** | Andersen Becser, N. and R. Hansen (2014). "Changes in levetiracetam plasma concentrations during pregnancy and its affection on seizure frequency." Epilepsia 55: 129. | Andersen B | 1 | 0 | 1 | 1 | 0 | No PK parameters | Levetiracetam |
| **8** | Arrive, E., et al. (2010). "Maternal and nenonatal tenofovir and emtricitabine to prevent vertical transmission of HIV-1: tolerance and resistance." Aids 24(16): 2478-2485. | Arrive | 1 | 0 | 1 | 0 | 0 | No PK parameters | Tenofovir and emtricitabine |
| **9** | Asymbekova, G. U. (1995). "A prospective study of antipyrine pharmacokinetics in pregnancy. [Russian]Prospektivnoe izuchenie farmakokinetiki antipirina pri beremennosti." Akusherstvo i ginekologiia(2): 19-22. | Asymbekova | 1 | 1 | 1 | 0 | 0 | No comparison group | Antipirin |
| **10** | Asymbekova, G. U., et al. (1995). "Pharmacokinetics and pharmacodynamics of aspirin prophylactic doses in pregnant females at high risk of placental insufficiency. [Russian]." Experimental and Clinical Pharmacology 58(2): 35-39. | Asymbekova | 1 | 1 | 1 | 0 | 0 | No comparison group | Aspirin |
| **19** | Benaboud, S., et al. (2011). "Population pharmacokinetics of nevirapine in HIV-1-infected pregnant women and their neonates." Antimicrobial Agents & Chemotherapy 55(1): 331-337. | Benaboud | 1 | 1 | 1 | 0 | 0 | No comparison group | Nevirapine |
| **23** | Berman, W., Jr., et al. (1980). "Pharmacokinetics of inhibitors of prostaglandin synthesis in the perinatal period." Seminars in Perinatology 4(1): 67-72. | Berman | 0 | 1 | 0 | 0 | 0 | Review | PG inhibitors |
| **29** | Blomback, M., et al. (1998). "A pharmacokinetic study of dalteparin (Fragmin) during late pregnancy." Blood Coagulation and Fibrinolysis 9(4): 343-350. | Blomback | 1 | 1 | 1 | 0 | 0 | No comparison group | LMWH (dalteparin) |
| **32** | Bologa, M., et al. (1991). "Pregnancy-induced changes in drug metabolism in epileptic women." Journal of Pharmacology and Experimental Therapeutics 257(2): 735-740. | Bologa | 1 | 0 | 1 | 1 | 0 | No PK parameters | AED |
| **33** | Bossi, L., et al. (1979). "The monitoring of anti-epileptic drugs during pregnancy. Clinical significance of modifications in plasma levels of anti-epileptic drugs. Clinical experience with 30 patients. [French]. Monitorage Des Medicaments Antiepileptiques Pendant La Grossesse. Signification Clinique Des Modifications Des Niveaux Plasmatiques Des Medicaments Antiepileptiques. Experiences Cliniques Chez 30 Malades." Lyon Medical 242(19): 597-603. | Bossi | 1 | 1 | 1 | 0 | 1 | No comparison group | Barbiturate |
| **58** | Croci, L., et al. (2012). "Pharmacokinetic and safety of raltegravir in pregnancy." European journal of clinical pharmacology 68(8): 1231-1232. | Croci | 1 | 1 | 0 | 1 | 0 | case report | Raltegravir |
| **59** | Dam, M., et al. (1979). "Antiepileptic drugs: Metabolism in pregnancy." Clinical pharmacokinetics 4(1): 53-62. | Dam | 1 | 1 | 1 | 0 | 0 | No comparison group | Phenytoin carbamazepine phenobarbitone |
| **62** | de Oliveira Baraldi, C., et al. (2012). "Effect of type 2 diabetes mellitus on the pharmacokinetics of metformin in obese pregnant women." Clinical pharmacokinetics 51(11): 743-749. | De Oliviera | 1 | 1 | 1 | 0 | 0 | Comparison to reported data not stated where the data is from and not stated in the results section | Metformin |
| **71** | Flaherty, J. F., et al. (1983). "Pharmacokinetics of cefoxitin in patients at term gestation: lavage versus intravenous administration." American Journal of Obstetrics & Gynecology 146(7): 760-766. | Flaherty | 1 | 1 | 1 | 0 | 0 | 2 patients (case report) No comparison group | Cefoxitin |
| **86** | Hartikainen-Sorri, A. L., et al. (1987). "Pharmacokinetics of clonidine during pregnancy and nursing." Obstetrics & Gynecology 69(4): 598-600. | Hartikainen | 1 | 0 | 1 | 0 | 0 | PK parameters. No comparison group. "nursing"- how much time passed from the delivery | Clonidine |
| **106** | Jharap, B., et al. (2014). "Intrauterine exposure and pharmacology of conventional thiopurine therapy in pregnant patients with inflammatory bowel disease." Gut 63(3): 451-457. | Jharap | 1 | 0 | 1 | 1 | 0 | No PK parameters | Thiopurine (azathioprine, mercaptopurine) |
| **114** | Kuhnz, W., E. Jager-Roman, et al. (1983). "Carbamazepine and carbamazepine-10,11-epoxide during pregnancy and postnatal period in epileptic mothers and their nursed infants: Pharmacokinetics and clinical effects." Pediatric Pharmacology 3(3-4): 199-208. | Kohnz | 1 | 1 | 1 | 0 | 0 | only 2 patients /comparison group | Carbamazepine |
| **140** | Na-Bangchang, K., et al. (2005). "The pharmacokinetics and pharmacodynamics of atovaquone and proguanil for the treatment of uncomplicated falciparum malaria in third-trimester pregnant women." European journal of clinical pharmacology 61(8): 573-582. | Na-Bangcha | 1 | 1 | 1 | 0 | 0 | Pregnant women in Thailand Vs. Zambia (no non-pregnant comparison group) | Atovaquone and proguanil |
| **145** | Norris, L. A., et al. (2004). "Low molecular weight heparin (tinzaparin) therapy for moderate risk thromboprophylaxis during pregnancy. A pharmacokinetic study." Thrombosis and Haemostasis 92(4): 791-796. | Norris | 1 | 0 | 1 | 0 | 0 | No comparison group | LMWH(tinzaparin) |
| **156** | Peiker, G., et al. (1985). "Examinations on the pharmacokinetics of amipicillin in pregnant women suffering from a H-gestosis during the last trimenon of the pregnancy. [German].Untersuchungen Zur Pharmakokinetik Von Ampicillin Bei Schwangeren Mit Einer H-Gestose Im Letzten Trimenon Der Schwangerschaft." Pharmazie 40(7): 480-481. | Peiker | 1 | 1 | 1 | 0 | 0 | No comparison group | Ampicillin |
| **187** | Shekhtman, M. M. and Z. M. Akhtamova (1985). "Pharmacokinetics of ampicillin and cephalosporin in the bodies of pregnant women and parturients. [Russian]Osobennosti farmakokinetiki ampitsillina i tsefalosporinov v organizme beremennykh i rodil'nits." Akusherstvo i ginekologiia(1): 9-11. | Shekhtman | 0 | 1 | 1 | 0 | 0 | Review and not a study | Ampicillin and cephalosporin |
| **125** | Shere, M., et al. (2014). "Optimizing periconceptional folic acid supplementation: Steady-state folate pharmacokinetics in pregnancy." FASEB Journal 1). | Shere M | 1 | 0 | 1 | 1 | 0 | PK parameters | Folic acid |
| **209** | von Mandach, U., et al. (1989). "Pharmacokinetic studies on fenoterol in maternal and cord blood." American journal of perinatology 6(2): 209-213. | Von Mandach | 1 | 1 | 1 | 0 | 0 | comparison to pregnant males | Fenoterol |
| **225** | Yu, T., et al. (2016). "Pregnancy-induced changes in the pharmacokinetics of caffeine and its metabolites." Journal of clinical pharmacology 56(5): 590-596. | Yu | 1 | 1 | 1 | 1 | 0 | Women were given coffee and not the drug | Caffeine |
| **226** | Shere, M., et al. (2015). "Pregnancy-induced changes in the long-term pharmacokinetics of 1.1 mg vs. 5 mg folic acid: a randomized clinical trial". J Clin Pharmacol 55(2):159-67. | Shere M | 1 | 0 | 1 | 0 | 0 | PK parameters, No comparison group | Folic acid |
| **229** | Colbers, A., et al. (2016). "Physiologically Based Modelling of Darunavir/Ritonavir Pharmacokinetics During Pregnancy." Clinical pharmacokinetics 55(3): 381-396. | Colbers | 0 | 0 | 0 | 0 | 0 | Mathematical model | Darunavir |
| **230** | Cressey, TR., et al. "Pharmacokinetics and virologic response of zidovudine/lopinavir/ritonavir initiated during the third trimester of pregnancy". AIDS 10;24(14):2193-200. | Cressey | 1 | 1 | 1 | 0 | 0 | No comparison group | Lopinavir/ritonavir |
